# Mosaic composition of *rib*A and *wsp*B genes flanking the *vir*B8-D4 operon in the *Wolbachia* supergroup B-strain, *w*Str

**DOI:** 10.1007/s00203-015-1154-8

**Published:** 2015-09-23

**Authors:** Gerald D. Baldridge, Yang Grace Li, Bruce A. Witthuhn, LeeAnn Higgins, Todd W. Markowski, Abigail S. Baldridge, Ann M. Fallon

**Affiliations:** Department of Entomology, University of Minnesota, 1980 Folwell Ave., St. Paul, MN 55108 USA; Department of Biochemistry, Molecular Biology and Biophysics, University of Minnesota, Minneapolis, MN 55455 USA; Feinberg School of Medicine, Northwestern University, Chicago, IL 60611 USA

**Keywords:** *Wolbachia*, LC–MS/MS, Proteomics, Mosaic genes, T4SS, RibA, RibB, WspB

## Abstract

The obligate intracellular bacterium, *Wolbachia pipientis* (*Rickettsiales*), is a widespread, vertically transmitted endosymbiont of filarial nematodes and arthropods. In insects, *Wolbachia* modifies reproduction, and in mosquitoes, infection interferes with replication of arboviruses, bacteria and plasmodia. Development of *Wolbachia* as a tool to control pest insects will be facilitated by an understanding of molecular events that underlie genetic exchange between *Wolbachia* strains. Here, we used nucleotide sequence, transcriptional and proteomic analyses to evaluate expression levels and establish the mosaic nature of genes flanking the T4SS *vir*B8-D4 operon from *w*Str, a supergroup B-strain from a planthopper (Hemiptera) that maintains a robust, persistent infection in an *Aedes albopictus* mosquito cell line. Based on protein abundance, *rib*A, which contains promoter elements at the 5′-end of the operon, is weakly expressed. The 3′-end of the operon encodes an intact *wsp*B, which encodes an outer membrane protein and is co-transcribed with the *vir* genes. WspB and vir proteins are expressed at similar, above average abundance levels. In *w*Str, both *rib*A and *wsp*B are mosaics of conserved sequence motifs from *Wolbachia* supergroup A- and B-strains, and *wsp*B is nearly identical to its homolog from *w*CobU4-2, an A-strain from weevils (Coleoptera). We describe conserved repeated sequence elements that map within or near pseudogene lesions and transitions between A- and B-strain motifs. These studies contribute to ongoing efforts to explore interactions between *Wolbachia* and its host cell in an in vitro system.

**Electronic supplementary material:**

The online version of this article (doi:10.1007/s00203-015-1154-8) contains supplementary material, which is available to authorized users.

## Introduction

*Wolbachia pipientis* (*Rickettsiales*; *Alphaproteobacteria*) is an obligate intracellular bacterium that infects filarial nematodes and a wide range of arthropods including ≥60 % of insects and ≈35 % of isopod crustaceans, but does not infect vertebrates (Hilgenboecker et al. 2008). *Wolbachia* is considered to be a single species classified into clades by multilocus sequence typing and designated as supergroups A to N (Baldo et al. 2006b; Comandatore et al. 2013; Lo et al. 2007). The C- and D-strains that infect filarial worms have phylogenies concordant with those of nematode hosts, consistent with strict vertical transmission as obligate mutualists (Comandatore et al. 2013; Dedeine et al. 2003; Li and Carlow 2012; Strubing et al. 2010; Taylor et al. 2005; Wu et al. 2004). Although arthropod-associated A- and B-strains may provide subtle fitness benefits to hosts (Zug and Hammerstein 2014), they are best known as reproductive parasites, causing phenotypes that maintain or increase *Wolbachia* infection frequencies, including feminization, parthenogenesis, and cytoplasmic incompatibility (Saridaki and Bourtzis 2010; Werren et al. 2008). Interference with host immune mechanisms and replication of arboviruses, bacteria and malarial plasmodia (Kambris et al. 2009; Pan et al. 2012; Zug and Hammerstein 2014) has encouraged efforts to exploit *Wolbachia* for biocontrol of arthropod vectors of vertebrate pathogens and/or crop pests (Bourtzis 2008; Rio et al. 2004; Sinkins and Gould 2006; Zabalou et al. 2004). An understanding of molecular differences between A- and B-strains, and how they have been influenced by horizontal transmission and genetic exchange (Newton and Bordenstein 2011; Schuler et al. 2013; Werren et al. 2008; Zug and Hammerstein 2014) will facilitate manipulation of *Wolbachia*.

*Wolbachia’s* interaction with host cells likely involves the type IV secretion system (T4SS), a macromolecular complex that transports DNA, nucleoproteins and “effector” proteins across the microbial cell envelope into the host cell, where they mediate intracellular interactions (Alvarez-Martinez and Christie 2009; Zechner et al. 2012). Homologs of all genes except *vir*B5 of *Agrobacterium tumefaciens* T4SS have been identified in *Wolbachia* and other members of the *Rickettsiales* (Gillespie et al. 2009, 2010), including *Anaplasma*, *Ehrlichia*, *Neorickettsia*, *Orientia and Rickettsia.* Among sequenced *Wolbachia* genomes, T4SS genes are organized in two operons: *vir*B3-B6 containing *vir*B3, *vir*B4 and four *vir*B6 paralogs and *vir*B8-D4 containing *vir*B8, *vir*B9, *vir*B10, *vir*B11, *vir*D4 and, in some genomes, the *wsp*B paralog of the *wsp*A major surface antigen (Pichon et al. 2009; Rances et al. 2008). In the supergroup B-strain *w*Pip from *Culex pipiens* mosquitoes, *wsp*B is disrupted by a transposon and is presumably inactive (Sanogo et al 2007). T4SS effector proteins that manipulate host cells have been identified from *Anaplasma* and *Ehrlichia* (Liu et al. 2012; Lockwood et al. 2011; Niu et al. 2010), and *Wolbachia* express both *vir* operons in ovaries of arthropod hosts, wherein T4SS effectors are suspected to play a role in cytoplasmic incompatibility and other reproductive distortions (Masui et al. 2000; Rances et al. 2008; Wu et al. 2004). Although WspA and WspB are likely components of the *Wolbachia* outer membrane, their functions remain unknown. In the case of *w*Bm, WspB is excreted/secreted into filarial host cells (Bennuru et al. 2009) and co-localizes with the Bm1_46455 host protein in tissues that include embryonic nuclei (Melnikow et al. 2011). WspB is therefore itself a candidate T4SS effector that may play a role in reproductive manipulation of the host.

The *Wolbachia* strain *w*Str in supergroup B causes strong cytoplasmic incompatibility in the planthopper, *Laodelphax striatellus* (Noda et al. 2001a), and in addition maintains a robust, persistent infection in a clonal *Aedes albopictus* mosquito cell line, C/*w*Str1 (Fallon et al. 2013; Noda et al. 2002). Because in vitro studies with *w*Str provide advantages of scale and ease of manipulation for exploring mechanisms that may facilitate transformation and genetic manipulation of *Wolbachia*, we have undertaken proteomics-based studies that provide strong support for expression of T4SS machinery in cell culture. Here, we report the sequence of the *vir*B8-D4 operon, including flanking genes *rib*A, upstream of *vir*B8, and *wsp*B downstream of *vir*D4. We show that *wsp*B is intact, describe protein structure predicted from the deduced WspB sequence, and verify co-transcription of *wsp*B with upstream *vir* genes. Relative abundance levels of WspB and the VirB8-D4 proteins in *w*Str are well above average, while RibA is among the least abundant of MS-detected proteins. In *w*Str, *rib*A and *wsp*B are mosaics of sequence motifs that are differentially conserved in supergroup A- (WOL-A) and B- (WOL-B) strains, and they contain conserved 8-bp repeat elements that may be associated with genetic exchange. Finally, we discuss implications for functional integration of the *Wolbachia* T4SS with WspB and with the riboflavin biosynthesis pathway enzymes GTP cyclohydrolase II (RibA) and dihydroxybutanone phosphate synthase (RibB).

## Materials and methods

### Cultivation of cells

*Aedes albopictus* C7-10 and C/*w*Str1 cells were maintained in Eagle’s minimal medium supplemented with 5 % fetal bovine serum at 28–30 °C in a 5 % CO_2_ atmosphere (Fallon et al. 2013; Shih et al. 1998). Cells were harvested during exponential growth, under conditions favoring maximal recovery of *Wolbachia* (Baldridge et al. 2014).

### Polymerase chain reaction, cloning and DNA sequencing

The polymerase chain reaction (PCR) was used to amplify *w*Str genes from DNA extracts prepared from *Wolbachia* enriched by fractionation of C/*w*Str1 cells on sucrose density gradients and recovered from the interface between 50 and 60 % sucrose (Baldridge et al. 2014). Template DNA was used to obtain 21 PCR products using a panel of 31 primers (Table S1), GoTaq™ DNA polymerase (Promega, Madison, WI), and a Techne TC-312 cycler (Staffordshire, UK). Cycle parameters were: 1 cycle at 94 °C for 2 min, 35 cycles at 94 °C for 35 s, 53 °C for 35 s, 72 °C for 1 min, followed by 1 cycle at 72 °C for 5 min. Extension time was increased to 2 min for products ≥1000 bp. PCR products were cloned in the pCR4-TOPO vector with the TOPO-TA Cloning Kit for Sequencing (Life Technologies, Grand Island, NY), and two or more clones each were sequenced at the University of Minnesota BioMedical Genomics Center.

### Reverse transcriptase polymerase chain reaction

Total RNA was purified from *A. albopictus* C7-10 and C/*w*Str1 cells using the PureLink RNA Mini Kit (Life Technologies) and treated with DNase I (RNase-free; Life Technologies) followed by heat inactivation, as suggested by the manufacturer. RT-PCR was executed with primers *vir*D4_F1764–1784_ and *wsp*B_R152–172_ (Table S1) using the RNA PCR Core Kit (Life Technologies) as suggested by the manufacturer with the exception that synthesized cDNA was treated with DNase-inactivated RNaseA before the final PCR reaction. The PCR reaction included 1 cycle at 95 °C for 4 min, 35 cycles at 95 °C for 35 s, 56 °C for 40 s, 72 °C for 40 s, followed by 1 cycle at 72 °C for 3 min. Reaction products were electrophoresed on 1 % agarose gels, cloned, and sequenced as above.

### Sequence alignments and protein structure prediction

DNA and protein sequence alignments were executed with the Clustal Omega program (Sievers et al. 2011). Alignments were edited by visual inspection and modified in Microsoft Word. WspB protein structure predictions were obtained using tools available at www.predictprotein.org, including the PROFtmb program (Dell et al. 2010) for prediction of bacterial transmembrane beta barrels (Bigelow et al. 2004) and per-residue prediction of up-strand, down-strand, periplasmic loop and outer loop positions of residues. The PROFisis program (Ofran and Rost 2006) was used to predict WspB amino acid residues that are potentially involved in protein–protein interactions. Trees were produced using PAUP* version 4 (Swofford 2002). Amino acids were aligned with Clustal W, using pairwise alignment parameters of 25/0.5 and multiple alignment parameters of 10/0.2 for gap opening and gap extension, respectively. The protein weight matrix was set to Gonnet. The alignment was saved as a nexus file and loaded into PAUP*, and the trees were created using a heuristic search with the criterion set to parsimony. Bootstrap 50 % majority-rule consensus trees are based on 1000 replicates, with *w*Bm (WOL-D) as the outgroup.

### Mass spectrometry, peptide detection, protein identification and statistical analysis

Mass spectrometry data, generated using LC–MS/MS on LTQ and Orbitrap Velos mass spectrometers as four data sets, were described previously (Baldridge et al. 2014). The MS search database was modified to include deduced ORFs from *w*Str sequence data described herein. All tests of association were performed with SAS version 9.3 (Cary, NC; http://www.sas.com/en_us/home.html/).

## Results

### Structure of the *w*Str *vir*B4-D8 operon

**Fig. 1:**
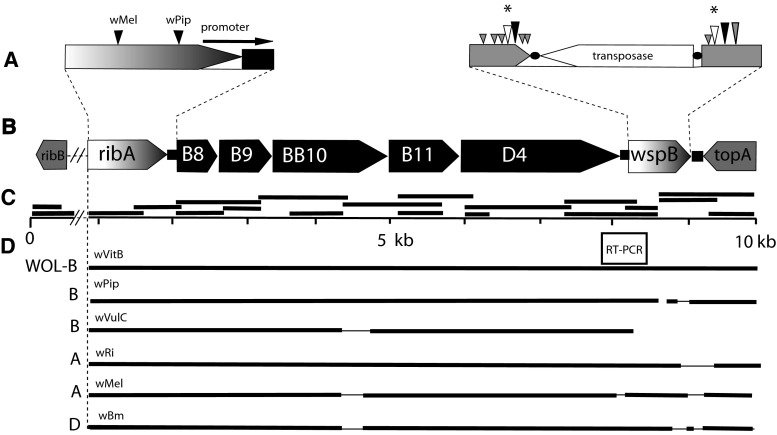
Schematic map of the *Wolbachia* T4SS *vir*B8-D4 operon and cloning strategy for the *rib*A to *top*A sequence from ^B^ *w*Str. **a** *Left* expanded view of the ^B^ *w*Str *rib*A ORF depicted as an *arrow* showing the direction of transcription. *Black horizontal arrow* indicates a putative promoter that extends into an intergenic spacer (*black rectangle*). *Black arrowheads* indicate positions of MS-detected unique peptides (95 % confidence). Gradient shading from *white* to *black* designates 5′-sequence identity resembling WOL-A transitioning to 3′-sequence more closely resembling WOL-B-strains. **a** *Right* expanded view of the interrupted *wsp*B homolog in ^B^ *w*Pip. *Black ellipses* indicate positions of IS256 inverted repeat elements flanking a 1.2-kb insertion encoding a MULE domain superfamily transposase (gi|190571636; pfam10551) on the opposite strand (indicated by the direction of the *open arrow*); flanking *gray shading* indicates *wsp*B. *Tall vertical black and gray arrowheads* indicate positions of unique peptides (95 and 94 % confidence, respectively) identified in the original MS data search. *Small gray arrows* indicate 95 % confidence peptides matched in a refined data set (including the ^B^ *w*Str sequence described here) that are conserved in WOL-B-strains, and *open arrowheads with stars* indicate peptides unique to ^B^ *w*Str. **b** Schematic depiction of the *Wolbachia* *vir*B8-D4 operon and flanking genes with *arrows* designating the direction of transcription. *Vir* genes are designated in *white font on a black background*; *black squares* indicate intergenic spacers. *Gradient shading* indicates mosaic structure of an intact wspB in ^B^ *w*Str. **c** *Filled lines* above the 10-kb scale marker represent cloned PCR amplification products (see Table S1 for primers) that were sequenced and assembled into the ^B^ *w*Str *rib*B and *rib*A–*top*A consensus sequence. The *double slash symbols at left* indicate that *rib*B is not contiguous with downstream genes. The *open box* indicates the RT-PCR amplification product from Fig. 2. **d** BLASTn alignment of the 9133-bp ^B^ *w*Str *rib*A–*top*A sequence to corresponding sequences in ^B^ *w*VitB ^B^ *w*Pip, ^B^ *w*VulC, ^A^ *w*Ri, ^A^ *w*Mel and ^D^ *w*Bm genomes. *Dark filled lines* indicate sequence identity >70 %; *light lines* indicate low sequence identity, and the open space in ^B^ *w*Pip represents an alignment gap

The robust, persistent infection of *A. albopictus* mosquito cell line, C/*w*Str1 with ^B^*w*Str (in the text below, strain designations are denoted by superscripts), isolated from the planthopper *L. striatellus*, provides an in vitro model to identify proteins that modulate the host–microbe interaction. A potential role for the T4SS is supported by strong representation of peptides from VirB8, VirB9, VirB10, VirB11, VirD4 (Table 1) and associated proteins in the ^B^*w*Str proteome (Baldridge et al. 2014). Despite its emergence as a useful strain that grows well in vitro, the ^B^*w*Str genome is not yet available. In *Wolbachia* strains for which genome annotation is available, gene order within the *vir*B8-D4 operon is conserved. Based on transcriptional analyses in the related genera, *Anaplasma* and *Ehrlichia* (Pichon et al. 2009), the promoter likely maps within the 3′-end of *rib*A extending into the intergenic spacer (Fig. 1a, black horizontal arrow at left) and is followed by five consecutive *vir* genes (Fig. 1b). In ^B^*w*Pip from *Culex pipiens* mosquitoes, *wsp*B is disrupted by insertion of an IS256 element that encodes a transposase on the opposite strand (Fig. 1a, at right; Sanogo et al. 2007). Because VirB8-D4 proteins were highly similar to homologs from ^B^*w*Pip (Baldridge et al. 2014), we evaluated *wsp*B in ^B^*w*Str and its potential expression as a *vir*B8-D4 operon member, as is the case in ^A^*w*Mel and ^A^*w*Ri from *Drosophila spp.* and ^A^*w*Atab 3 from the wasp *Asobara tabida* (Rances et al. 2008; Wu et al. 2004). In the original proteomic analysis, three WspB peptides (Fig. 1a, tall black and gray arrows represent 95 and 94 % confidence peptides, respectively) mapped proximal and distal to the transposon insertion in ^B^*w*Pip, while the absence of peptides corresponding to the transposon suggested that *wsp*B is intact in ^B^*w*Str.

**Table 1:** MS-detected peptides from *w*Str proteins encoded by *rib*A, *rib*B and the *vir*B8-D4 operon

Protein	^a^kDa	^b^Pep(1)	^b^Pep(2)	^b^Pep(T)	^c^Cov.	^d^RAL	^e^SR
RibA	41	2	2	2	6	0.5	−2.30
RibB	24	7	12	12	89	7.0	1.20
VirB8	26	9	10	10	58	5.0	0.59
VirB9	31	10	8	10	45	6.2	0.84
VirB10	54	14	16	18	53	8.8	0.94
VirB11	37	12	14	14	42	7.0	0.82
VirD4	77	12	14	14	26	6.2	0.45
WspB	31	2^f^	11	50	7.2	1.08	

^a^Protein mass in kilodaltons. ^b^ Number of 95 % confidence unique peptides; (1) designates original search [7]; (2) designates a refined search in which the database included peptides based on the present *w*Str nucleotide sequence data; (T) combined total peptides from both searches. ^c^ Percent protein sequence coverage represented by detected peptides. ^d^ Mean number of peptides from four independent MS data sets. ^e^ Studentized residual based on the modified univariable model of the refined search (Table S3, column R); SR value 0 indicates average abundance protein, 0–1 above average, 1–2 abundant and >2 highly abundant. Values below 0 indicate lower than average abundance. ^f^ A 94 % confidence peptide indicated in Fig. 1A did not meet the threshold for proteome inclusion in the original search. For VirB10, one originally detected peptide was absent from the refined search

### Nucleotide and deduced amino acid sequence comparisons

To examine the *vir*B4-D4 operon in ^B^*w*Str, we sequenced overlapping PCR products from 20 primer pairs (Table S1) spanning 9.1 kb beginning 43 bp downstream of the 5′-end of *rib*A in other *Wolbachia* strains and ending within *top*A encoded immediately downstream of the operon on the opposite strand (Fig. 1b, c). With the notable exception of the ^B^*w*Pip transposon, the nucleotide sequence aligned most closely to homologous sequences from ^B^*w*VitB and ^B^*w*Pip. In addition, we noted variability in an ~0.3-kb region of *vir*B10 in ^B^*w*Str that was conserved in ^B^*w*VitB, ^B^*w*Pip and ^A^*w*Ri, but not in ^B^*w*VulC, ^A^*w*Mel and ^D^*w*Bm (Fig. 1d; see Table S2 for GenBank Accessions).

Pairwise sequence comparisons of the *vir*B8-D4 operon from ^B^*w*Str to homologs from *Wolbachia* supergroup A, B, C, D and F strains (Table 2) confirm that *vir*B10, with nucleotide identities ranging from 74–99 %, is the least conserved of the five *vir* genes, and we note that Klasson et al. (2009) attributed divergence of *vir*B10 in ^A^*w*Mel and ^A^*w*Ri to genetic exchange with a WOL-B-strain. Collectively and as individuals, the *vir* genes from ^B^*w*Str have the highest nucleotide identities (~99 %) with ^B^*w*VitB and ^B^*w*Pip. Identities with five A-strains are lower (range 87–91 %), lower yet (range 80–89 %) with the F-strain, ^F^*w*Cle and fall to a range of 74–88 % with three nematode-associated strains, ^D^*w*Bm, ^C^*w*Oo and ^C^*w*Ov. At the 5′-end of the operon, *rib*A was distinct, with approximately equivalent nucleotide identity with homologs from A- and B-strains (range 91–94 %), while the partial sequence of *top*A downstream of the operon had a conservation pattern similar to that of the *vir* genes. In some comparisons, *vir*B8, *vir*B11, *vir*D4 and *top*A amino acid identities exceed nucleotide identities. Although *ribB* is not physically adjacent to the *vir*B8-D4 operon in annotated *Wolbachia* genomes, *rib*B from ^B^*w*Str is most similar to homologs from ^B^*w*No (97 % nucleotide identity) and ^A^*w*Mel (90 %), but was exceptional because identities with three other insect-associated A- and B-strains (~80 %) were lower than with F-, C- and D-strains (range 85–87 %). Consistent with earlier proteomic data (Baldridge et al. 2014), in all comparisons that discriminate between A- and B-strains, ^B^*w*Str resembled WOL-B, while variability in *rib*A and *wsp*B flanking the *vir*B8-D4 genes exceeded that of the *vir* genes themselves.

**Table 2:** Pairwise nucleotide and amino acid comparisons

Gene	^B^ *w*PiP		^B^ *w*VitB		^B^ *w*No		^B^wTai		^B^ *w*VulC		^A^ *w*Mel		^A^ *w*Ri	
	N	AA	N	AA	N	AA	N	AA	N	AA	N	AA	N	AA
*rib*A^a^	94	89	94	89	93	88	94	90	93	92	93	91	92	89
*vir*B8	*99*	*100*	*99*	*100*	*99*	*99*	*99*	*100*	94	94	88	86	88	87
*vir*B9	*99*	*99*	*99*	*98*	*98*	*97*	*97*	*97*	94	93	91	89	91	89
*vir*B10	*99*	*99*	*99*	*98*	90	86	*98*	96	88	74	87	74	88	85
*vir*B11	*99*	*99*	*97*	*99*	96	*98*	*97*	*99*	90	93	89	95	89	95
*vir*D4	*99*	*99*	*99*	*99*	*99*	*99*	*99*	*99*	94	*97*	89	92	89	93
*wsp*B	56	xx	*98*	96	85	68	–	–	–	–	85	70	85	70
*top*A^a^	*99*	*100*	*99*	*100*	*99*	*99*	–	–	–	–	88	87	87	86
*ribB* ^a^	81	80	–	–	*97*	96	–	–	–	–	90	91	79	78

Gene	^A^ *w*Ana		^A^ *w*Kue		^A^ *w*Atab3		^F^ *w*Cle		^D^ *w*Bm		^C^ *w*Oo		^C^wOv	
	N	AA	N	AA	N	AA	N	AA	N	AA	N	AA	N	AA
*rib*A^a^	91	88	93	91	–	–	84	81	83	80	82	74	82	75
*vir*B8	88	87	88	86	88	88	85	83	85	81	83	81	84	82
*vir*B9	91	89	91	89	91	89	84	84	84	84	82	76	81	76
*vir*B10	88	84	87	74	87	73	80	71	84	70	76	64	74	64
*vir*B11	89	95	89	95	89	95	89	95	88	94	86	89	87	89
*vir*D4	89	93	89	93	88	92	87	92	87	87	86	91	88	94
*wsp*B	83	68	85	70	85	70	72	xx	73	61	72	49	71	49
*top*A^a^	86	85	–	–	–	–	88	92	86	88	84	83	84	88
*ribB* ^a^	80	88	–	–	–	–	87	87	86	87	85	xx	85	xx

*Wolbachia* strains from supergroups A, B, C, D and F are indicated by superscripts, with percentages of nucleotide (N) and amino acid (AA) sequence identities to ^B^ *w*Str. Dashes indicate sequences not available, and xx indicates pseudogenes; GenBank Accession numbers are given in Table S2

^a^Partial gene and protein sequences: *rib*A 1040 bp, *rib*B 592 bp; *top*A 825 bp. Host associations: *w*Pip, *Culex pipiens*—mosquito; *w*VitB, *Nasonia vitripennis*—wasp; *w*Tai, *Teleogryllus taiwanensis*—cricket; *w*VulC, *Armadillidium* *vulgare*—isopod; *w*Mel, *w*Ri, *w*Ana, *w*No, *Drosophila* spp.—fruit fly; *w*Kue, *Ephestia kuehniella*—moth; *w*Atab 3 *Asobara tabida*—wasp; *w*Bm, *w*Oo and *w*Ov from filarial nematodes *Brugia malayi*, *Onchocerca ochengi* and *O. volvulus,* respectively. In the comparison, values of 97 % or greater are shown in italics

### Expression and relative abundances of the ^B^*w*Str *vir*B4-D8 proteins

To refine an earlier original proteomic analysis (Baldridge et al. 2014), we incorporated the PCR-amplified ^B^*w*Str sequences described here to the database for peptide identification [Table 1, see column labeled Pep(2)]. Statistical analysis indicated that in a univariable model, protein molecular weight was weakly (*r*^2^ = 0.2221) but significantly (*p* < 0.0001) associated with peptide count: log(*peptides*) = −0.40247 + 0.4953 × log(*MW*). Estimations of protein relative abundance levels (RAL) based on peptide counts were therefore normalized to protein length using studentized residuals (SR), a measure of deviance from expected values adjusted for estimated SD from the mean. All peptide data and SR values in the univariable and multivariable models of the original and refined searches are detailed in Table S3.

In the refined search, we identified eight new peptides from Vir proteins [Table 1, compare columns labeled Pep(2) to Pep(1)], including three from the most divergent VirB10. In aggregate, the five Vir proteins had a mean (SD) SR of 0.73 (0.2) and are expressed at above average abundance. We identified five new peptides from RibB, but none from RibA (Table 1). RibB has an SR of 1.2 and is an abundant protein, while RibA has an SR of −2.3 and is among the least abundant of MS-detected proteins. Nine new peptides from the highly divergent WspB (see below) generated an SR of 1.08, slightly above the threshold (>1.0) for an abundant protein and roughly equivalent to SR values (range 1–1.17) of housekeeping proteins such as isocitrate dehydrogenase, ftsZ, ATPsynthase F0F1 α subunit, and ribosomal proteins S2, S9, L3, L7/L12 and L14 (Table S3). In comparison, WspA with an SR of 2.17 (Table S3, entry 63) ranked as highly abundant, and the most abundant protein in the proteome was the GroEL chaperone (entry 586), with an SR of 3.66.

### Reverse transcriptase PCR confirms co-transcription of *wsp*B with vir genes

**Fig. 2:**
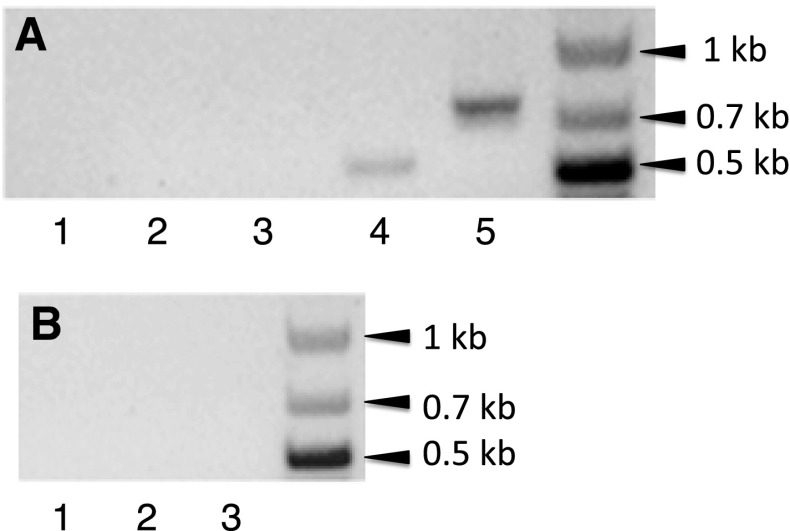
Reverse transcriptase PCR (RT-PCR) analysis shows co-transcription of *wsp*B with *vir*D4. **a** *Lanes 1 and 2* RT-PCR negative controls with no RNA or with no reverse transcriptase, respectively. *Lanes 3 and 4* RT-PCR of RNA from uninfected C7-10 and infected C/*w*Str1 cells, respectively, with *vir*D4 forward and *wsp*B reverse primers. *Lane 5* RT-PCR positive control with C/*w*Str1 RNA and *Wolbachia* primers S12F/S7R, which amplify portions of a ribosomal protein operon described previously (Fallon 2008). **b** *Lane 1* PCR negative control with no Taq enzyme. *Lanes 2 and 3* negative control lacking RT, with RNA from uninfected C7-10 and infected C/*w*Str1 cells, respectively

Similar SR values for WspB, relative to VirB8-D4, were consistent with evidence that *wsp*B is co-transcribed with *vir*B8-D4 in ^A^*w*Mel, ^A^*w*Ri and ^A^*w*Atab 3 (Rances et al. 2008; Wu et al. 2004). We used RT-PCR with RNA template verified by PCR to be free of DNA contamination (Fig. 2b, lanes 2 and 3) to amplify a 528-bp product that was produced in reactions containing RNA from C/*w*Str1 cells (Fig. 2a, lane 4), but not in negative control reactions (lanes 1 and 2) or those with RNA from C7-10 cells (lane 3). Its sequence matched the expected ^B^*w*Str genomic sequence (Fig. 1c, RT-PCR box at right), confirming that in ^B^*w*Str*, wsp*B is a member of the *vir*B8-D4 operon.

### In ^B^*w*Str, *rib*A is a mosaic of conserved WOL-A and WOL-B sequence motifs

**Fig. 3:**
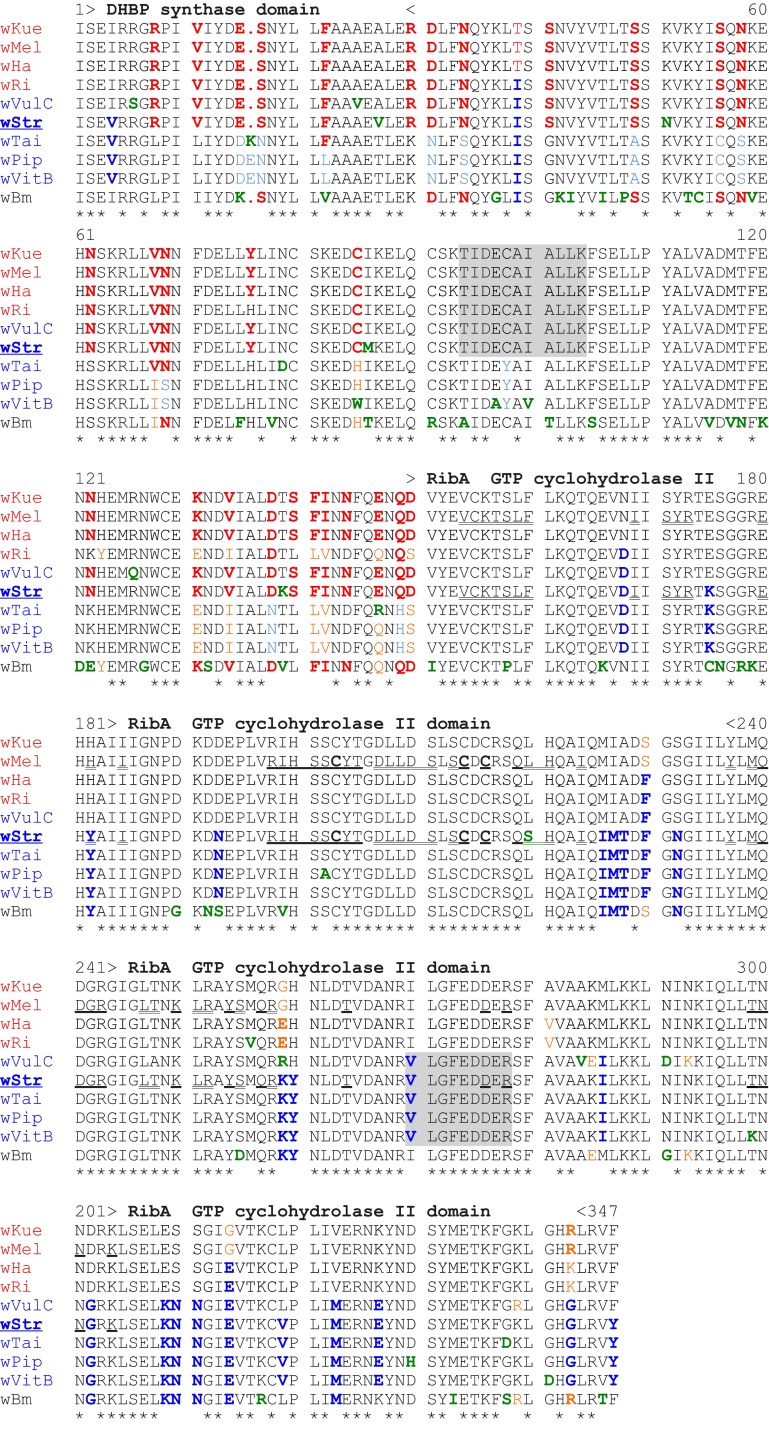
Amino acid sequence alignment of RibA homologs from ^*B*^ *w*Str and *Wolbachia* supergroups A (*red*), B (*blue*) and D (*black*) respectively. *Asterisks below the alignment* indicate universally conserved residues. Unique residues are in *green font*. Residues conserved in ^B^ *w*Str and a majority of B-strains are in *dark blue*, *bold font*, while those in *dark red*, *bold font* are conserved with a majority of A-strains. Residues conserved in two to four strains are in *light blue*, *orange* or *orange bold font*. Residues highlighted in *gray* correspond to 95 % confidence peptides detected by LC–MS/MS. The dihydroxybutanone phosphate synthase (RibB) and GTP cyclohydrolase II domains (RibA) are indicated above the alignment within *greater than* *less than* symbols. *Bold underlined* residues in ^A^ *w*Mel and ^B^ *w*Str indicate conserved active site amino acids, including critical cysteine residues. *Double underlined* residues indicate amino acids involved in the dimerization interface. See Tables 2 and S2 for host associations and GenBank Accessions. The PCR-amplified ^B^ *w*Str sequence does not encode the N-terminal amino acids; position 1 corresponds to the 15th amino acid

The *rib*A nucleotide sequence has been shown to contain regulatory elements for expression of the T4SS operon in *Anaplasma* and *Ehrlichia* (Ohashi et al. 2002; Pichon et al. 2009). In contrast to highest homologies of ^B^*w*Str *vir*B8-D4 genes to WOL-B-strains, *rib*A sequence identities showed little difference between WOL-A and -B homologs (Table 2), but the two MS-detected peptides corresponded to ^A^*w*Mel and ^B^*w*Pip homologs, respectively (Fig. 1a). Alignment of amino acids from 10 RibA homologs (Fig. 3; WOL-A and WOL-B-strains are identified at left in red and blue, respectively) suggested that ^B^*w*Str RibA is a two-part mosaic, each containing a protein functional domain.

**Fig. 4:**
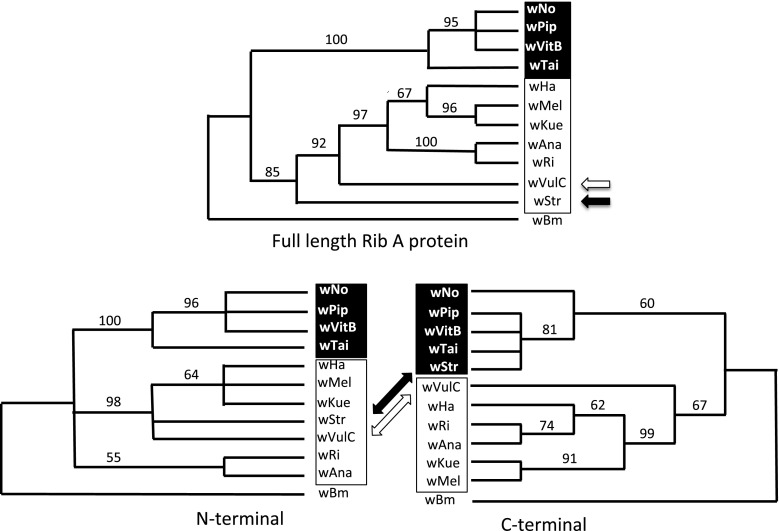
Phylogenic relationships of ^B^ *w*Str RibA protein with homologs from WOL-A- and WOL-B-strains. Consensus trees show bootstrap values based on 1000 replicates, with ^D^ *w*Bm (WOL-D) as the outgroup. WOL-A-strains are shown in *black font boxed* against a *white background*. WOL-B-strains are shown in *white font on a black background*. *Open arrows* designate ^B^wVulC and *closed arrows* indicate ^B^ *w*Str. The N-terminal alignment corresponded to the first 150 residues in Fig. 3; the remainder of the protein was included in the C-terminal alignment

The amino terminal 150 residues in ^B^*w*Str RibA (Fig. 3) include a short dihydroxybutanone phosphate synthase domain and the first detected peptide (residues 94–104). This portion of ^B^*w*Str RibA matched sequences from the four A-strains and a single B-strain, ^B^*w*VulC, at 29 of 36 variable amino acids (shown in red), while only three (4, 39 and 168 in blue) matched the other three B-strains and four (in green) were unique. In contrast, the C-terminal 151–347 residues, encompassing the second peptide (residues 250–258) within a GTP cyclohydrolase domain, included a single amino acid unique to ^B^*w*Str, while 23 (in blue) uniformly matched B-strains except ^B^*w*VulC, which continued to resemble the A-strains until residue 239. Among the four A-strains, the ^B^*w*Ri homolog is most similar throughout the alignment to the B-strains, but within residues 129–150 immediately preceding the cyclohydrolase domain, it closely matched ^B^*w*Tai, ^B^*w*Pip and ^B^*w*VitB, while ^B^*w*Str and ^B^*w*VulC matched the other three A-strains. In aggregate, the alignment suggested that the ^B^*w*Str and ^B^*w*VulC homologs are two-part mosaics, each containing a protein functional domain, with an N-terminal WOL-A motif and a C-terminal WOL-B motif. We note that the C-terminal B-strain motif is consistent with the B-strain identity of the downstream *vir*B8-D4 operon (Table 2) and includes the predicted promoter region (Ohashi et al. 2002; Pichon et al. 2009). Likewise, in a phylogenetic comparison (Fig. 4), trees representing the full length and N-terminal regions (top and bottom left) show ^B^*w*VulC and ^B^*w*Str in adjacent positions, and grouped more closely with WOL-A-strains. In the C-terminus, where the amino acid alignment shows an overall higher consensus (Fig. 3), ^B^*w*Str grouped with the B-strains including ^B^*w*Pip, while ^B^*w*VulC appears more closely related to A-strains.

### Nucleotide alignment and phylogenetic comparisons show that *rib*A is a mosaic gene in ^B^*w*Str and ^B^*w*VulC

A nucleotide alignment (Fig. S1) confirmed that *ribA* from ^B^*w*Str is a two-part mosaic of WOL-A and WOL-B sequence motifs that correspond to the N- and C-terminal halves of the protein. In the first 522 nucleotides of *ribA*, 45 (in red font) of 56 variable nucleotides in ^B^*w*Str match the A-strain sequences (Fig. S1), but only six (in blue) match the majority of B-strains and two are unique to ^B^*w*Str (in green). In the downstream 522 nucleotides of *rib*A, 51 (in blue) of 54 variable nucleotides in ^B^*w*Str match B-strains, while a single nucleotide (684 in red) matches the A-strains and two (in green) are unique to ^B^*w*Str. In ^B^*w*VulC, *rib*A has a similar two-part mosaic structure but does not firmly transit from the WOL-A to the WOL-B sequence motif until position 775, consistent with the amino acid alignment. Among the A-strains, *rib*A from ^A^*w*Ri is again most similar to the B-strain sequences. Within nucleotides 387–453 encoding amino acids 129–150 just before the cyclohydrolase domain and the A/B-strain sequence motif transition in ^B^*w*Str, 13 of 18 WOL-A/B variable nucleotides in ^A^*w*Ri are shared with ^B^*w*Tai, ^B^*w*Pip and ^B^*w*VitB, but those of ^B^*w*Str and ^B^*w*VulC are conserved with the other A-strains (orange and black vs. red residues, respectively).

### WspB in ^B^*w*Str is strikingly similar to a ^A^*w*CobU4-2 homolog

**Fig. 5:**
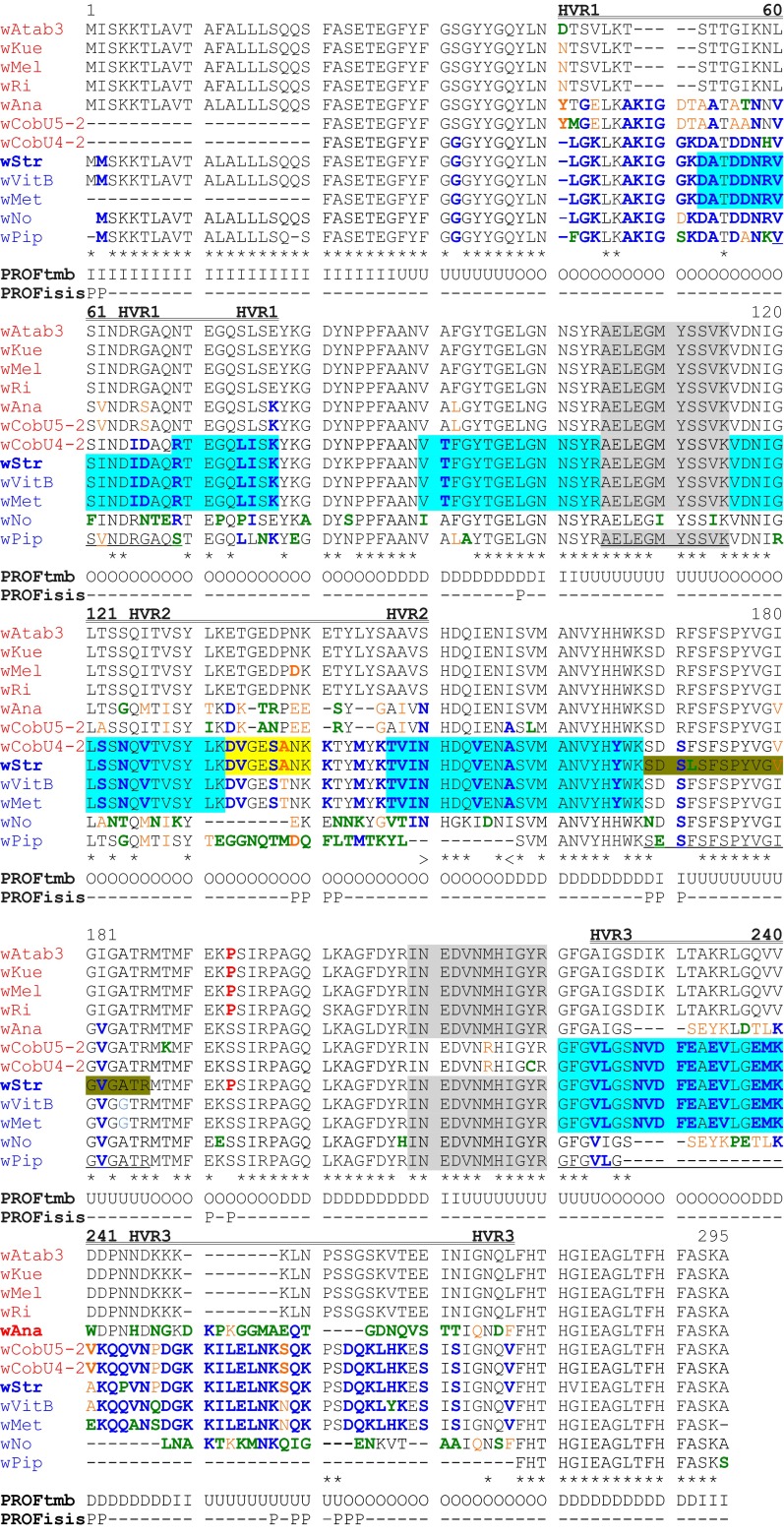
Amino acid sequence alignment of WspB homologs. At *left, font color* designates WOL-A (*red*) and B (*blue*) strains, and the ^B^ *w*Str sequence is the top listed Wol-B-strain. *Asterisks below alignment indicate* universally conserved residues; three hypervariable regions (HVRs) are *doubly underlined above the alignment*. *Blocks of coloring* designate peptides detected by LC–MS/MS at the 95 % confidence level. Those in *gray* were conserved in A- and B-strains. *Cyan* designates peptides conserved in B-strains, and *yellow*, those conserved in ^B^ *w*Str and ^A^ *w*CobU4-2. *Olive* peptides were unique to ^B^ *w*Str. Residues conserved between ^B^ *w*Str and a majority of A-strains are in *red font* (a single proline at residue 193) and residues conserved with a majority of B-strains are in *blue font*. Unique residues are in *green font*, and residues conserved between two or three homologs are in *orange font*. *Underlined residues below the alignment* denote the breakpoints between contiguous peptides within sequence regions. The *greater than* and *less than* symbols below the alignment indicate a transposon insertion in the *wsp*B pseudogene of ^B^ *w*Pip, followed by two additional deduced ORFs—see Fig. S2. PROFtmb (prediction of transmembrane beta barrels) symbols for individual residues below the alignment are: U—up-strand, D—down-strand, I—periplasmic loop, O—outer loop. PROFisis (prediction of protein–protein interaction residues) symbol P designates interaction residues. *Wolbachia* strain host associations: ^A^ *w*Atab 3, *A. tabida*—wasp; ^A^ *w*Cob, *C. obstrictus*—weevil; ^B^ *w*Met, *Metaseiulus occidentalis*—predatory mite. See Tables 2 and S2 for other host associations and GenBank Accessions. The first 20 residues of the^A^ *w*Cob and ^B^ *w*Met sequences are not available

Having shown that *wsp*B is intact in ^B^*w*Str, we mapped 11 peptides onto amino acid sequences encoded by 12 homologs (Fig. 5), including sequences deduced from three open reading frames (ORFs) in the *wsp*B pseudogene from ^B^*w*Pip (Sanogo et al. 2007) and two overlapping ORFs in a pseudogene from ^A^*w*CobU4-2, one of several WOL-A variants associated with the weevil, *Ceutorhynchus obstrictus*. Of two ^B^*w*Str peptides (Fig. 5) detected at 95 % confidence in the original search (Baldridge et al. 2014), the first (residues 105–115 in gray) was identical in all strains except ^B^*w*No, which has unique M/I and V/I substitutions (residues in green). The second peptide (residues 209–220) is identical in all but the two ^A^*w*Cob strains that share an M/R substitution (215 in orange), while ^A^*w*CobU4-2 has a unique Y/C substitution (219 in green). Five additional ^B^*w*Str peptides (highlighted in cyan) were identical with ^B^*w*VitB and ^B^*w*Met (residues in blue), but not with ^B^*w*Pip and ^B^wNo, which have many residues that are unique (in green) or shared (in orange) only with ^A^*w*CobU5-2 and ^A^*w*Ana. Thus, with the exception of ^A^*w*CobU5-2, cyan peptides of ^B^*w*Str match other WOL-B-strains.

Two peptides underscore a striking similarity between the ^B^*w*Str and ^A^*w*CobU4-2 homologs. The first (Fig. 5, residues 133–140 highlighted in yellow) contains an alanine residue (138 in bold orange) shared only with ^A^*w*CobU4-2. The second (residues 169–186 highlighted in olive) has a unique F/L substitution (in green) and a V/I substitution (in orange) shared with ^A^*w*CobU4-2 and ^A^*w*Ana. Overall, the ^B^*w*Str and ^A^*w*CobU4-2 sequences differ at only five residues (59, 172, 193, 215 and 219), of which four occur within hypervariable regions. Throughout the alignment, ^A^*w*Atab 3, ^A^*w*Kue, ^A^*w*Mel and ^A^*w*Ri form a conserved group, but the divergent ^A^*w*Ana and ^A^*w*CobU4-2 and U5-2 strains have multiple residues (in blue, as in 42–77 and 224–277) that are conserved with the B-strains, suggesting genetic exchange between supergroups.

### WspB domain structure and hypervariable regions (HVRs)

WspB is a paralog of the better-known WspA major surface antigen, which is anchored in the cell envelope by a transmembrane β-barrel domain (Koebnik et al. 2000), while surface-exposed loop domains contain HVRs with high recombination frequencies within and between strains (Baldo et al. 2010). The PROFtmb program predicted 10 transmembrane down (D)- and up (U)-strands and six periplasmic space (I) strands in WspB from ^B^*w*Str (Fig. 5; residues indicated by D, U and I, respectively; *Z* score of 6.8 supports designation as transmembrane β-barrel protein). HVR1 and HVR2 each contain a predicted outer loop (residues 38–86 and 115–156 indicated by O) with high proportions of amino acids that are potentially charged at physiological pH; HVR3 contains two outer loops. Finally, a small predicted loop that is not within an HVR contains a proline (residue 193) that is conserved in ^B^*w*Str and four WOL-A-strains. It is one of the 20 amino acids, most with hydrophilic or potentially charged side chains and within HVRs or adjacent to periplasmic space strands, predicted by the PROFisis program to be potentially involved in protein–protein interactions (P below alignment).

#### HVR1 amino acids

In HVR1 (Fig. 5, residues 41–77), eight residues are universally conserved among all homologs, while the majority of variable residues are differentially conserved in the B-strains (residues in blue) versus the A-strains. However, the sequences from the ^A^*w*Ana and ^A^*w*CobU5-2 A-strains are mosaics in which eight of the first 20 residues (in blue) are conserved with all B-strains, while eight others are either conserved mutually or with ^B^*w*No or ^B^*w*Pip (in orange). Within the remaining 17 residues of HVR1, the ^A^*w*Ana and ^A^*w*CobU5-2 sequences are better conserved with the other A-strains, while ^B^*w*No and ^B^*w*Pip have multiple unique residues (in green). The ^A^*w*CobU4-2 and ^B^*w*Str sequences differ only at residue 59.

#### HVR2 amino acids

Within HVR2 (Fig. 5, residues 121–150), ^A^*w*CobU5-2 and ^A^*w*Ana sequences have alignment gaps at four residues, five or six unique residues respectively (in green), and eight residues that are either conserved mutually (in orange) or with ^B^*w*No. The ^B^*w*Pip pseudogene has only the first two residues of HVR2 due to a transposon insertion (indicated below alignment by *greater than less than *symbols). The ^A^*w*CobU4-2 pseudogene contains a nucleotide sequence duplication (see below) that results in an overlap of the first and third ORFs beginning at the seventh residue of HVR2, but their spliced sequences, as shown, are identical to that of ^B^*w*Str. The ^B^*w*No sequence has eight alignment gaps and nine unique residues.

#### HVR3 amino acids

In HVR3, five of 52 residues (Fig. 5, residues 224–277) are conserved among all strains. Throughout HVR3, sequences from the upper cluster of four A-strains are identical, including an alignment gap. However, the ^A^*w*Ana sequence has 22 unique residues (in green) and is partially conserved with ^B^*w*No (nine residues in orange). In striking contrast to differences in HVR1 and HVR2, the ^A^*w*CobU4-2 and U5-2 homologs have identical HVR3 sequences that are conserved with the B-strains, particularly ^B^*w*Str (residues in blue), differing only at residues 241 and 244.

### Nucleotide sequence alignment confirms a mosaic *wsp*B and identifies a conserved repeated sequence

Nucleotide sequence alignment of eleven *wsp*B homologs confirmed that WOL-A/B genetic mosaicism is concentrated in the HVR regions and revealed three copies of a repeated sequence element within or near HVR2. Further analyses identified three copies of the repeated sequence element in *rib*A at the 5′-end of the *vir*B8-D4 operon and four copies in *vir* genes.

#### HVR1

HVR1 (Fig. S2, nucleotides 117–241) from ^B^*w*Str begins with two nucleotides (117 and 120 in red) that are conserved in ^B^*w*Str and all WOL-A-strains except ^A^*w*CobU5-2 and ^A^*w*CobU4-2. Downstream, the ^B^*w*Str sequence includes 47 of 48 nucleotides (in blue) within a sequence motif characteristic of ^B^*w*Str and the other B-strains. The ^A^*w*CobU5-2 and ^A^*w*Ana sequences are initially similar to the WOL-B motif, but beginning at an alignment gap in the other A-strains they have 11 nucleotides (in orange, nucleotides 152–207) that are conserved with ^B^*w*No and ^B^*w*Pip at positions in which those strains diverge from the WOL-B consensus. Thus, HVR1 in ^B^*w*Str begins with nucleotides from a conserved WOL-A sequence motif but transitions to the conserved WOL-B motif, while HVR1 from the ^A^*w*CobU4-2 A-strain differs from that WOL-B motif at a single nucleotide (176). In contrast, the ^A^*w*Ana and ^A^*w*CobU5-2 sequences are mosaics of the WOL-A and WOL-B consensus motifs and share nucleotides with the divergent ^B^*w*No and ^B^*w*Pip B-strains, which also closely resemble each other upstream of HVR1 (23 nucleotides in light blue and one in orange).

#### HVR2 contains conserved repeat elements

HVR2 (Fig. S2, nucleotides 361–450) contains a conserved WOL-B sequence motif that differs at 20 nucleotides (in blue), from the WOL-A motif, while the divergent sequences from ^B^*w*No, ^B^*w*Pip, ^A^*w*Ana and ^A^*w*CobU5-2 share an alignment gap and are again similar (nucleotides in orange). A tandem repeated sequence at nucleotides 365–379, **CAAGTAA**T**CAAGTAAC**, in the B-strains ^B^*w*Str, ^B^*w*VitB and ^B^*w*Met occurs with slight variation (underlined residues) as **CAAGTA**G**CCAA**A**TAAC**, in the A-strains ^A^*w*Atab 3, ^A^*w*Kue, ^A^*w*Mel and ^A^*w*Ri. We designated the eight-bp sequence, CAARTARY, where R = A or G, and Y = C or T, as an HVR2-repeat. The pseudogene from ^A^*w*CobU4-2 contained a third copy of CAAGTAAT that interrupted ORF1 and was removed from the alignment (indicated by upwards arrow below alignment) to shift to ORF3, which maintains identity to the deduced amino acid sequence from ^B^*w*Str. Just downstream of HVR2 at nucleotides 457–463, a truncated copy of the HVR2-repeat lacking the 3′-terminal pyrimidine is conserved in ^B^*w*Str, ^B^*w*VitB, ^B^*w*Met and ^A^*w*CobU4-2 and corresponds to the position (indicated by *greater than less than* symbols below alignment) of the transposon insertion in ^B^*w*Pip. Finally, we noted that the most divergent HVR2 sequences from ^A^*w*Ana, ^A^*w*CobU5-2, ^B^*w*No and ^B^*w*Pip have T/C and A/G substitutions (in orange, light blue and green) that disrupt the HVR2-repeat consensus.

#### HVR3

Within HVR3 (Fig. S2, nucleotides 670–831), conserved sequence motifs occur in the upper cluster of four A-strains and in the B-strains (nucleotides in blue), with the exceptions of ^B^*w*Pip (HVR3 absent) and ^B^*w*No. Sequences from ^A^*w*CobU4-2 and ^A^*w*CobU5-2 are identical despite their major differences in HVR1 and HVR2 and differ from the B-strain consensus only at nucleotides 722 and 773 (in orange). The ^A^*w*Ana and ^B^*w*No sequences are the most divergent but share 43 variable nucleotides (in orange) and have 67 and 18 unique residues (in green), respectively.

### HVR2-repeats also occur in *rib*A and *rib*B

Based on a DNA pattern search (http://bioinformatics.org/sms/), three HVR2-repeats occur in *rib*A, two in *vir*D4, and single copies in *vir*B8 and *vir*B9 (Table 3). In addition, a reverse complement of the CAARTARY sequence occurs at the same position in *rib*B from three WOL-A-strains and ^B^*w*Pip (see gray shading in Fig. S3). The ^B^*w*Pip homolog contains a second copy at residues 7–14 just downstream of the start codon (not shown) and is a WOL-A/B mosaic (see below). Although repeat frequencies in individual *rib*A (0.29) and *wsp*B (0.34) genes are ~sixfold higher than in the whole genomes of ^A^*w*Mel and ^B^*w*Pip (0.05) from flies (Diptera), it will be important to re-evaluate these frequencies when a ^B^*w*Str genome (Hemipteran host) becomes available.

**Table 3:** Distribution of HVR2-repeats in ^B^ *w*Str *vir*B8-D4 operon and genomes of ^A^ *w*Mel and ^B^ *w*Pip

Repeat	*rib*A	*vir*B8	*vir*B9	*vir*D4	*wsp*B	*w*Mel	*w*Pip
CAAGTAAT/C	118/145	–	–	−5943^b^	7610/7618	154	239
CAAATAAT/C	672	–	−2485	−5919^b^	–	275	360
CAAGTAGC	–	1288	–	–	7702^b^	187	104
Total	3	1	1	2	3	616	703
Frequency^a^	0.29	0.15	0.13	0.12	0.34	0.05	0.05

Values indicate 5′-nucleotide positions of HRV2-repeats in the 9133-bp *rib*A to *top*A sequence from ^B^ *w*Str (see Fig. 1; Acc. KF43064.1). Negative values indicate reverse complement positions. Copy numbers in the complete ^A^ *w*Mel (NC_002978.6) and ^B^ *w*Pip (NC_010981.1) genomes are shown at right

^a^Frequency is defined as number repeats/total nucleotides in each individual gene (or complete genome) indicated at the top of the panel, ×100

^b^See underlined nucleotides 457–463 in Fig. S2, which lack the 3′-terminal pyrimidine

Although RibA and RibB are involved in riboflavin biosynthesis, *rib*B is not contiguous with *rib*A and the *vir*V8-D4 operon, and it has higher variability than *rib*A (Table 2). Among the WOL-B-strains, *rib*B in ^B^*w*Str and ^B^*w*No is conserved with the ^A^*w*Au and ^A^*w*Mel A-strains (Fig. S3; note especially the bold blue residues downstream of nucleotide 181, as well as additional residues in orange). In contrast, the ^B^*w*Pip homolog is best-conserved (nucleotides in red) with WOL-A-strains, ^A^*w*Ana, ^A^*w*Ha and ^A^*w*Ri, including an alignment gap at residue 483 encompassing an identical 15-nucleotide “island” with the reverse complement CAARTARY repeat. Downstream of the gap, at residue 511, the ^B^*w*Pip sequence shifts to a predominantly WOL-B motif conserved in ^B^*w*Str, ^B^*w*No, but also in ^A^*w*Mel (nucleotides in blue), while ^A^*w*Ana, ^A^*w*Ri and ^A^*w*Ha are mutually conserved (nucleotides in orange) versus all other strains. Within the 3′-end of the alignment (nucleotides 541–600), the ^B^*w*Pip sequence is conserved with ^B^*w*Str, ^B^*w*No and ^D^*w*Bm (nucleotides in blue), while ^A^*w*Au and ^A^*w*Mel are the most divergent (nucleotides in green).

## Discussion

Although the status of *Wolbachia* as a species remains unclear (Baldo et al. 2006b; Lo et al. 2007), a notable distinction between WOL-C-/D-strains that associate with nematodes as mutualists and WOL-A-/B-strains that occur as reproductive parasites in insects relates to genome stability and phylogenetic congruence between *Wolbachia* and its host. In insect hosts, *Wolbachia* appears to engage in frequent horizontal gene transfer, resulting in a lack of phylogenetic congruence manifested by gene structures that represent mosaic recombinations from genomes now considered distinct strains. Coinfections with two or more *Wolbachia* strains and activities of bacteriophages that reside in genomes of WOL-A/B-strains likely contribute to this genetic plasticity (Bordenstein and Reznikoff 2005; Newton and Bordenstein 2011), which may reflect what some authors suggest is a worldwide *Wolbachia* pandemic (Zug et al. 2012). Examples of natural coinfections include ^A^*w*AlbA and ^B^*w*AlbB in *A. albopictus* mosquitoes (O’Neill et al. 1997), ^A^*w*VitA and ^B^*w*VitB in the parasitoid wasp, *N.vitripennis* (Perrot-Minnot et al. 1996; Raychoudhury et al. 2008) and ^A^*w*Ha and ^B^*w*No in the phytophagous *D. simulans* (James et al. 2002). A particularly interesting example in *C. obstrictus* weevils involves infection with a single ^A^*w*Cob strain, in which polymorphisms in *wsp*A and *wsp*B indicate that three distinct variants coexist in all host populations (Floate et al. 2011) and it will be of interest to explore other genetic similarities and differences among these variants following separation in vitro and/or in uninfected hosts. *Wolbachia* coinfections have also been documented in insects such as fig wasps (Yang et al. 2012), tephritid flies (Morrow et al. 2014) and planthoppers (Zhang et al. 2013) whose interactions with parasitoids, parasites and predator arthropods may facilitate horizontal transmission (Cordaux et al. 2001; Werren et al. 2008; Zug et al. 2012). In nature, the ^B^*w*Str strain occurs in two planthopper hosts (Noda et al. 2001a) and in the strepsipteran endoparasite *Elenchus japonicus* (Noda et al. 2001b; Zhang et al. 2013). In the present study, ^B^*w*Str has been artificially introduced into a cultured cell line, which has not been achieved with ^B^*w*Pip or nematode-associated strains. Adaptation of ^B^*w*Str to cell lines (Noda et al. 2002; Fallon et al 2013) will provide an in vitro system for examining mechanisms of genetic exchange if conditions for maintenance of doubly infected cells can be developed through coinfection or somatic cell fusion. We note that high rates of recombination and transposition in *Wolbachia* (Baldo et al. 2006a; Cordaux et al. 2008) are consistent with expression of an abundant RecA protein (SR 1.05; Table S3, entry 146) as well as 18 transposases and/or proteins with transposase domains in ^B^*w*Str (Baldridge et al. 2014).

### Genetic plasticity of *wsp*B in the *vir*B8-D4 operon

An intact *wsp*B that maps to the 3′-end of the *vir*B8-D4 operon in most WOL-A genomes (Wu et al. 2004) is absent from 17 of 21 WOL-B-strains, including ^B^*w*VulC and nearly all other isopod-associated strains (Pichon et al. 2009), and is interrupted by a transposon in ^B^*w*Pip (Sanogo et al. 2007). Here, we verify that in ^B^*w*Str, an intact *wsp*B is co-transcribed with *vir*D4 and is expressed in C/*w*Str1 cells as an abundant protein at levels similar to those of many housekeeping proteins. The *wsp*B structure closely resembles that of its better-studied *wsp*A paralog, encoding a major surface antigen that has four HVR regions with sequence motifs that have been shuffled by recombination within and between *Wolbachia* WOL-A- and -B-strains (Baldo et al. 2005, 2010). Likewise, most sequence variation in *wsp*B alleles occurs in the three HVR regions, with distinctive patterns for each region. HVR1 underscores WOL-A/B mosaicism in ^A^*w*Ana and ^A^*w*CobU5-2, and in addition it shows a high level of identity between ^A^*w*CobU4-2 and ^B^*w*Str. Similarity between ^A^*w*Ana and ^A^*w*CobU5-2 and between ^B^*w*Str and ^A^*w*CobU4-2 also occurs in HRV2, while ^B^*w*No stands out as distinctive. In ^B^*w*Pip, HVR2 is disrupted by a transposon insertion and we identified an eight-nucleotide HRV2-repeat (CAARTARY) that correlates with transitions between WOL-A-/B-strain motifs and the pseudogene lesions in ^B^*w*Pip and ^A^*w*CobU4-2. Finally, we noted that high identity of ^A^*w*CobU5-2, ^A^*w*CobU4-2 and ^B^*w*Str is unique to HVR3.

The remarkable similarity of the *wsp*B homologs from ^B^*w*Str and ^A^*w*CobU4-2 (>98 % nucleotide identity Fig. S2) is consistent with exchange of an apparently intact gene between members of distinct *Wolbachia* supergroups by a mechanism that requires further investigation. Intensive analysis of the *wsp*A paralog demonstrates that intragenic recombination breakpoints are concentrated in conserved regions outside of the HVRs (Baldo et al. 2005, 2010). CAARTARY repeats are not present in *wsp*A, and in *wsp*B, they occur only within and directly adjacent to HVR2 at positions that correspond to pseudogene lesions in ^A^*w*CobU4-2 and in ^B^*w*Pip (due to a transposition event in ^B^*w*Pip; Sanogo et al. 2007). Furthermore, Pichon et al. (2009) suggested that transposition events may explain absence of *wsp*B in the *vir*B8-D4 operons of many WOL-B-strains. In a practical sense, CAARTARY repeats at *wsp*B pseudogene lesions and WOL-A/B sequence motif transitions (Figs. S1, S2, S3) suggest their involvement in genetic exchange. Because transformation of *Wolbachia* has not yet been achieved, engineering of CAARTARY repeats into vectors used successfully to introduce selectable markers into other members of the *Rickettsiales* (see Beare et al. 2011) merits investigation.

### Potential functions of WspB

Although bacterial outer membrane proteins are important mediators of interactions with host cells and specific function(s) of both WspA and WspB remain to be identified, they may have unique functions as porin proteins in *Wolbachia*, which lack cell walls. The *vir*B8-D4 operons of *Wolbachia* and its sister genera, *Anaplasma* and *Ehrlichia*, are similarly organized (Gillespie et al. 2010; Hotopp et al. 2006) with 3′- terminal genes encoding major surface proteins that, analogous to *wsp*B, are co-transcribed with the *vir* genes (Ohashi et al. 2002). In *A. marginale*, a family of *msp*2 pseudogenes undergo “combinatorial gene conversion” at the expression site (Brayton et al. 2002) and MSP2 variants change during growth in different host cell types, which likely reflects a response to host immunity mechanisms (Chávez et al. 2012). Similarly, Baldo et al. (2010) proposed that changes in WspA HVR regions play a role in host adaptation and innate immunity interactions, consistent with variation in the higher-order structure of the protein in different hosts (Uday and Puttaraju 2012). HVR sequence changes in the *wsp*B paralog may reflect a similar dynamic. Additional evidence indicates that MSP2 proteins are glycosylated (Sarkar et al. 2008), which is now an established process in post-translational modification in bacteria (Dell et al. 2010; Nothaft and Szymanski 2010), and we note that WspB contains potential glycosylation sites. Although an inactivated pseudogene or absence of *wsp*B in *vir*B8-D4 operons of some *Wolbachia* strains indicates that it is not absolutely required for survival, a secretome analysis of *Brugia malayi* showed that WspB from ^D^*w*Bm is excreted/secreted into filarial host cells (Bennuru et al. 2009). Furthermore, it co-localizes with the Bm1_46455 host protein in tissues that include embryonic nuclei (Melnikow et al. 2011). WspB is therefore itself a candidate T4SS effector that may play a role in reproductive manipulation of the host. Mosaicism in *wsp*B and its high rate of evolution (Comandatore et al. 2013) may thus reflect genetic changes that optimize adaptation to particular host cells such as those in reproductive tissues and facilitate exploitation of new arthropod niches by *Wolbachia*.

### Genetic plasticity of *rib*A in the *vir*B8-D4 operon

Aside from *wsp*B at the 3′-end of the T4SS *vir*B8-D4 operon, *rib*A exhibits genetic plasticity at its 5′-end. In both ^B^*w*Str and ^B^*w*VulC, *rib*A is a two-part mosaic of N-terminal WOL-A and C-terminal WOL-B motifs. In contrast, the internal *vir*B8-D4 genes have typical B-strain identities, and in some strain comparisons, amino acid identities slightly exceed nucleotide identities, which Pichon et al. (2009) attribute to strong selection against non-synonymous codon substitutions. Among the internal *vir*B8-D4 genes, however, Klasson et al. (2009) suggest that in ^A^*w*Ri, an especially variable region in *vir*B10 is likely derived from genetic exchange with a B-strain. We note here that *rib*A from ^A^*w*Ri closely resembles B-strain homologs within a variable region that immediately precedes the GTP cyclohydrolase domain, where its homolog in ^B^*w*Str transitions from WOL-A to WOL-B sequence motifs (Fig. S1, positions 387–450).

In contrast to ^D^*w*Bm, in which *rib*A and *vir*B8 are co-transcribed and bind common transcription factors (Li and Carlow 2012), relative abundance levels suggest that in ^B^*w*Str, *rib*A is transcribed independently of the *vir*B8-D4 operon. Some WOL-B-strains, such as ^B^*w*VulC, lack *wsp*B at the 3′-terminus of the *vir*B8-D4 operon, while our data confirm that in ^B^*w*Str, *wsp*B is co-transcribed with the *vir* genes, consistent with similar relative abundances of WspB and the five Vir proteins. In aggregate, these observations suggest that WOL-D and WOL-A-/B-strains may differ in how RibA and WspB expression interfaces with T4SS-mediated transport of effectors in filarial worms and arthropod hosts (Felix et al. 2008; Masui et al. 2000; Rances et al. 2008; Wu et al. 2004), and it will be of interest to explore whether such differences relate to riboflavin provisioning. In filarial nematodes (Li and Carlow 2012; Strubing et al. 2010; Wu et al. 2009) and bedbugs (Hosokawa et al. 2010), evidence suggests that *Wolbachia* provisions host with riboflavin, the precursor of flavin cofactors that are essential for many cellular redox reactions. In contrast, riboflavin depletion reduces ^B^*w*Str abundance in C/*w*Str1 cells, suggesting that ^B^*w*Str utilizes host riboflavin and does not augment riboflavin levels in mosquito host cells (Fallon et al. 2014).

### Potential functions of RibA and RibB

In initial commitment steps in riboflavin biosynthesis, enzymatic activities encoded by the *rib*A and *rib*B functional domains use GTP and ribulose-5-phosphate as substrates to catalyze riboflavin biosynthesis, consuming 25 molecules of ATP per molecule of riboflavin (Bacher et al. 2000). We note that in *Wolbachia* genomes, *rib*A is the annotated homolog of *rib*BA in *Escherichia coli* (Brutinel et al. 2013) and encodes a dihydroxybutanone phosphate synthase domain with putative RibB function near the N-terminus, upstream of a GTP cyclohydrolase II domain with conserved dimerization and active site residues (RibA function). As in *E. coli*, *Wolbachia* genomes also encode *rib*B, but at a distinct chromosomal locus, suggesting that *rib*A and *rib*B are not coordinately expressed. In *Sinorhizobium meliloti* (*Rhizobiales*; *Alphaproteobacteria*), knockout mutations of *rib*BA decreased flavin secretion but did not cause riboflavin auxotrophy or block establishment of symbiosis, suggesting that RibBA may have an undefined role in molecular transport (Yurgel et al. 2014). As is the case with ^B^*w*Str, RibB is at least threefold more abundant than RibA in the bacterium *Acidithiobacillus ferrooxidans* (Knegt et al. 2008). In yeast, RibB has thiol-dependent alternative redox states (McDonagh et al. 2011), partially localizes to the mitochondrial periplasm, and has an unexplained function in oxidative respiration that is independent of riboflavin biosynthesis (Jin et al. 2003). These observations raise the possibility that in *Wolbachia*, RibA and RibB may have functions other than riboflavin biosynthesis that integrate with pathways involved in cellular oxidative state, such as iron metabolism. Intracellular bacteria are challenged by host-imposed oxidative stress and iron starvation (reviewed by Benjamin et al. 2010) and riboflavin biosynthesis is associated with iron acquisition in bacteria such as *Helicobacter pylori* (Worst et al. 1998) and *Campylobacter jejuni* (Crossley et al. 2007). *Wolbachia* interferes with iron metabolism and sequestration in insects (Brownlie et al. 2009; Kremer et al. 2009) and influences iron-dependent host processes such as heme metabolism, oxidative stress, apoptosis and autophagy (Gill et al. 2014). We note that the periplasmic iron-binding component of a membrane transporter is an abundant protein in ^B^*w*Str (Table S3, entry 778 and Baldridge et al. 2014).

## Electronic supplementary material

Table S1 Polymerase chain reaction primers and amplification products obtained from the ^B^ *w*Str genes, *rib*A, *rib*B, *vir*B8-D4, *wsp*B and *top*A. (DOCX 108 kb)

Table S2 Genbank accession numbers for all *Wolbachia* homologs of *rib*A, *rib*B, *vir*B8-D4, *wsp*B and *top*A, including those from ^B^ *w*Str. N/A: not applicable either because the sequences are not available or were not used in the comparisons reported in the tables and figures. The ^C^ *w*Ov genome is not annotated and the numerical values refer to genome coordinates determined by BLAST comparisons to ^B^ *w*Str. (XLSX 177 kb)

Table S3 Results of univariable and multivariable analyses after log transformation of the outcome, Peptide Count, and predictor, Molecular Weight. This table reports results for the refined search of the MS data sets with inclusion of sequences of cloned ^B^ *w*Str genes reported here and highlighted in yellow within the Table. Results of the original search of the four MS data sets were detailed previously (Baldridge et al. 2014). See tabs at bottom: Sheet 1 reports Mean SR values for all proteins in original and refined models in columns M and R; Univariable model and Multivariable Model (adjusted for functional class and MS Dataset) for results of tests of association. Runs 1, 2, 3 and 4 correspond to MS data sets D, E, F and G, respectively. (XLS 283 kb)

Figure S1 Nucleotide alignment of *rib*A homologs from ^*B*^ *w*Str and WOL-A, B- and D-strains at left in red, blue and black font, respectively. Nucleotides encoding the dihydroxybutanone phosphate synthase and GTP cyclohydrolase II domains are indicated above the alignment within *greater than* *less than* symbols. Asterisks below alignment indicate universally conserved nucleotides. Unique nucleotides are in green font. Nucleotides conserved in ^B^ *w*Str and a majority of B-strains are in dark blue bold font, while those in dark red bold font are conserved with a majority of A-strains. Nucleotides conserved in two to four strains are in light blue, orange or orange bold font. Nucleotides highlighted in gray and cyan indicate the MS-detected ^A^ *w*Mel and ^B^ *w*Pip 95% confidence peptides shown in Fig. 1, with amino acids indicated at top. Underlined nucleotides correspond to the CAARTARY repeat. See Tables 2 and S2 for host associations and Genbank Accessions. (DOCX 292 kb)

Figure S2 Nucleotide sequence alignment of *wsp*B homologs from ^B^ *w*Str and WOL-A and B-strains as indicated by red and blue font at left. Nucleotides conserved between ^B^ *w*Str and a majority of A-strains are in red font and residues conserved with a majority of B-strains are in blue font. Asterisks below alignment indicate universally conserved nucleotides and double underlines above the alignment indicate three hypervariable regions (HVRs). Unique nucleotides are in green font and residues conserved between two to four strains are in light blue, orange or bold orange font. The *greater than less than* symbols below alignment indicate a transposon insertion in the *wsp*B pseudogene of ^B^ *w*Pip, which is aligned as three discontinuous sequence blocks corresponding to nucleotides 1334165 - 1334594; 1335958 - 1336167; 1336271 – 1336326 from Accession NC_010981.1. The three CAARTARY repeats are underlined (nucleotides 365–379 and 457–463). Highlighted residues correspond to 95% confidence peptides detected by LC–MS/MS (amino acids indicated at top; lower case indicates additional matched peptides not unique to WspB) that were conserved in most strains (gray), conserved in B-strains (cyan), conserved in ^B^ *w*Str and ^A^ *w*CobU4-2 (yellow), or unique to ^B^ *w*Str (olive). See Tables 2 and S2 for host associations and Genbank Accessions. (DOCX 271 kb)

Figure S3 Nucleotide sequence alignment of *rib*B homologs from ^B^ *w*Str and WOL-A, B- and D-strains at left in red, blue and black font, respectively. Asterisks below the alignment indicate universally conserved nucleotides. Unique nucleotides are in green font. Nucleotides conserved in ^B^ *w*Str and a majority of B-strains are in dark blue bold font, while those in dark red bold font are conserved with a majority of A-strains. Nucleotides conserved in two to four strains are in light blue, orange or orange bold font. See Tables 2 and S2 for host associations and Genbank Accessions. (DOCX 250 kb)
