# Supplementary material for: Mosaic composition of ribA and wspB genes flanking the virB8-D4 operon in the Wolbachia supergroup B-strain, wStr
Source: Arch Microbiol. 2015 Sep 23;198:53–69. doi: 10.1007/s00203-015-1154-8 (PMC4705124; doi:10.1007/s00203-015-1154-8)
Supplement: Supplementary file 1 — Polymerase chain reaction primers and amplification products obtained from the B wStr genes, ribA, ribB, virB8-D4, wspB and topA. (DOCX 108 kb) [file 203_2015_1154_MOESM1_ESM.docx]

**Table S1.** Polymerase chain reaction primers and amplification products obtained from the ^B^*w*Str genes, *rib*A, *rib*B, *vir*B8-D4, *wsp*B and *top*A.

| Gene | Name | Forward primer | Name | Reverse primer | Product bp |
| --- | --- | --- | --- | --- | --- |
| *ribA* | GTPcyc-F1 | CCATCAGTGAAGTCAGGCGT | GTPcyc-R2 | ATGCCATTCCCAAAGTCGGT | 700 |
| *rib*A | GTPcyc-F3 | GATGTATATGAAGTGTGCAAAACAT | VirB8-R3 | G GCTCAATAGTGCTACTTGTGC | 858 |
| *rib*B | RibB-F1 | ATAAGTTTGTCGATGGTAGTTAAC | RibB-R1 | GGTATTTCTTCTGTGGAAGATG | 592 |
| *rib*B | RibB-F2 | CACCTGTTGTAATGCCATAACG | RibB-R1 | GGTATTTCTTCTGTGGAAGATG | 301 |
| *top*A | WspB-561F | ACCGTCAATAAGACCCGCAG | TopA-F2 | TCAAATGGACATGCAGATTGG | 1373 |
| *vir*B8 | VirB8-F1 | GATAAGGATATAAATTGGAATTCAA | VirB9-R2 | GCGACTACTGACTTCAAATGGC | 901 |
| *vir*B8 | VirB8-F1 | GATAAGGATATAAATTGGAATTCAA | VirB9-R1 | AGCCTTTTACAGGATTCGCCT | 1313 |
| *vir*B9 | VirB9-F1 | GCCATTTGAAGTCAGTAGTCGC | VirB9-R1 | AGCCTTTTACAGGATTCGCCT | 434 |
| *vir*B9 | VirB9-F1 | GCCATTTGAAGTCAGTAGTCGC | VirB10-R1 | ATCCATGGCAGTTAGCGCATC | 1571 |
| *vir*B9 | VirB9-F2 | ATTCGATGATGGTTATTTAACC | VirB10-R1 | ATCCATGGCAGTTAGCGCATC | 1225 |
| *vir*B10 | VirB10-F1 | AACTGATCTCTTGCCACCC | VirB10-R1 | ATCCATGGCAGTTAGCGCATC | 757 |
| *vir*B10 | VirB10-F2 | GCTGTTGTAGGACAAAAAGCCTC | VirB11-R2 | ATCAGGCTCACATGCTGGAG | 1594 |
| *vir*B11 | VirB11-F1 | CTACAGAACAAAAACTGAGCG | VirB11-R1 | AAGAGCCATTGCCGGACTATC | 632 |
| *vir*B11 | VirB11-F1 | CTACAGAACAAAAACTGAGCG | VirD4-R2 | GGAAAAGGTGTCAAGCTAGG | 997 |
| *vir*D4 | VirD4-F1 | TGTTGACGGTCCAGATGGTG | VirD4-R1 | CCAGAGCCTGTAGGTGCAAA | 403 |
| *vir*D4 | VirD4-F1 | TGTTGACGGTCCAGATGGTG | VirD4-R4 | TGATCATCACGTGGCAGCAT | 1492 |
| *vir*D4 | VirD4-F2 | CAAGAAATTATTATGCTGCCAC | WspB-80R | CCTTCTGTTTCACTTGCAAAAG | 639 |
| *wsp*B | VirD4-F2 | CAAGAAATTATTATGCTGCCAC | WspB-580R | CTGCGGGTCTTATTGACGGT | 1151 |
| *wsp*B | WspB-60F | CTTTTGCAAGTGAAACAGAAGG | WspB-580R | CTGCGGGTCTTATTGACGGT | 535 |
| *wsp*B | WspB-561F | ACCGTCAATAAGACCCGCAG | TopA-F1 | CGGATTCTATATAAAATGCGG | 657 |
| *vir*D4 | virD4_F1764-1784_ | AGAGAGTAATGCTATTGAAGC | wspB_R152-172_ | GGTTATCATCTGTAGCATCTT | 528 |
