# Supplementary material for: Mosaic composition of ribA and wspB genes flanking the virB8-D4 operon in the Wolbachia supergroup B-strain, wStr
Source: Arch Microbiol. 2015 Sep 23;198:53–69. doi: 10.1007/s00203-015-1154-8 (PMC4705124; doi:10.1007/s00203-015-1154-8)
Supplement: Supplementary file 4 — Nucleotide alignment of ribA homologs from B wStr and WOL-A, B- and D-strains at left in red, blue and black font, respectively. Nucleotides encoding the dihydroxybutanone phosphate synthase and GTP cyclohydrolase II domains are indicated above the alignment within greater than less than symbols. Asterisks below alignment indicate universally conserved nucleotides. Unique nucleotides are in green font. Nucleotides conserved in B wStr and a majority of B-strains are in dark blue bold font, while those in dark red bold font are conserved with a majority of A-strains. Nucleotides conserved in two to four strains are in light blue, orange or orange bold font. Nucleotides highlighted in gray and cyan indicate the MS-detected A wMel and B wPip 95% confidence peptides shown in Fig. 1, with amino acids indicated at top. Underlined nucleotides correspond to the CAARTARY repeat. See Tables 2 and S2 for host associations and Genbank Accessions. (DOCX 292 kb) [file 203_2015_1154_MOESM4_ESM.docx]

Nucleotide alignment of *rib*A homologs

1> **DHBP synthase domain** <60

wKue ATCAGCGAAA TCAGGCGCGG TC**GG**CCAATT **G**TAATATAT**- --**GATGAAA**G** TAA**T**TA**C**CTA

wMel ATCAGCGAAA TCAGGCGCGG TC**GG**CCAATT **G**TAATATAT**- --**GATGAAA**G** TAA**T**TA**C**CTA

wHa ATCAGCGAAA TCAGGCGCGG TC**GG**CCAATT **G**TAATATAT- --GATGAAA**G** TAA**T**TA**C**CTA

wRi ATCAGCGAAA TCAGGCGCGG TC**GG**CCAATT **G**TAATATAT**- --**GATGAAA**G** TAA**T**TA**C**CTA

wVulC ATCAGCGAAA T**T**AG**AA**G**T**GG CC**GG**CCAATT **G**TAATATAT**- --**GATGAAA**G** TAA**T**TA**C**CTA

**wStr** ATCAG**T**GAA**G** TCAGGCG**T**GG TC**GG**CCAATT **G**TAATATAT**- --**GATGAAA**G** TAA**T**TA**C**CTA

wTai ATCAG**T**GAA**G** TCAGGCG**T**GG TCTTCCAATT TTAATATATG ATGATAAAAA TAACTATCTA

wPip ATCAG**T**GAA**G** TCAGGCG**T**GG TCTTCCAATT TTAATATATG ATGATGAAAA TAACTATCTA

wVitB ATCAG**T**GAA**G** TCAGGCG**T**GG TCTTCCAATT TTAATATATG ATGATGAAAA TAACTATCTA

wBm ATCAGCGAAA T**T**AGGCGCGG CCT**A**CCAATT **A**TAATATAT- --GATAAAA**G** TAA**T**TATCTA

***** *** * ** * ** * ****** ******** *** *** *** ***

61 > **DHBP synthase domain** < 120

wKue TTG**T**TTGCTG CTGCTGA**GG**C TTTAGAAA**G**A **G**ATTTATTTA **A**TCAATA**C**AA GCTTACATCA

wMel TTG**T**TTGCTG CTGCTGA**GG**C TTTAGAAA**G**A **G**ATTTATTTA **A**TCAATA**C**AA GCTTACATCA

wHa TTG**T**TTGCTG CTGCTGA**GG**C TTTAGAAA**G**A **G**ATTTATTTA **A**TCAATA**C**AA GCTTACATCA

wRi TTG**T**TCGCTG CTGCTGA**GG**C TTTAGAAA**G**A **G**ATTTATTTA **A**TCAATA**C**AA GCTTA**T**ATCA

wVulC TTG**T**TTGCTG CTG**T**TGA**GG**C TTTAGAAA**G**A **G**ATTTATTTA **A**TCAATA**C**AA GCTTA**T**ATCA

**wStr** TTG**T**TTGCTG CTGCTGA**GGT** TTTAGAAA**G**A **G**ATTTATTTA **A**TCAATA**C**AA GCTTA**T**ATCA

wTai TTG**T**TTGCTG CTGCTGAAAC TTTAGAAAAA AATTT**G**TTTA GTCAATATAA GCTTA**T**ATCA

wPip TTGCTTGCTG CTGCTGAAAC TTTAGAAAAA AATTTATTTA GTCAATATAA GCTTA**T**ATCA

wVitB TTGCTTGCTG CTGCTGAAAC TTTAGAAAAA AATTTATTTA GTCAATATAA GCTTA**T**ATCA

wBm TTG**G**TTGCTG CTGC**C**GA**G**AC TTTAGAAAAA **G**A**C**TT**G**TTTA **A**TCA**G**TA**CGG** GCTTA**TC**TCA

*** ****** *** ** ******** * ** **** ****** ***** ***

121 180

wKue **A**GTAATGT**A**T ATGTTACTTT **A**ACT**T**CAAGT AAGGTAAAAT ACATAT**C**TCA GA**A**TAAAGAA

wMel **A**GTAATGT**A**T ATGTTACTTT **A**ACT**T**CAAGT AAGGTAAAAT ACATAT**C**TCA GA**A**TAAAGAA

wHa **A**GTAATGT**A**T ATGTTACTTT **A**ACT**T**CAAGT AAGGTAAAAT ACATAT**C**TCA GA**A**TAAAGAA

wRi **A**GTAATGT**A**T ATGTTACTTT **A**ACT**T**CAAGT AAGGTAAAAT ACATAT**C**TCA GA**A**TAAAGAA

wVulC **A**GTAATGT**A**T ATGTTACTTT **A**ACT**T**CAAGT AAGGTAAAAT ACATAT**C**TCA AA**A**TAAAGAA

**wStr** **A**GTAATGT**A**T ATGTTACTTT **A**ACT**T**CAAGT AA**C**GTAAAAT ACATAT**C**TCA GA**A**TAAAGAA

wTai GGTAATGT**A**T ATGTTACTTT GACTGCAAGT AAGGTAAAAT ACATATGTCA GAGTAAAGAA

wPip GGTAATGTGT ATGTTACTTT GACTGCAAGT AAGGTAAAAT ACATATGTCA GAGTAAAGAA

wVitB GGTAATGTGT ATGTTACTTT GACTGCAAGT AAGGTAAAAT ACATATGTCA GAGTAAAGAA

wBm GGTAA**GA**T**A**T ATGTTA**T**T**C**T **AC**CT**T**CAAGT AAGGTAA**CG**T **G**CATAT**C**TCA AA**ACGTG**GA**G**

**** * * ****** * * ** ***** ** **** * ***** *** * **

181 240

wKue CATA**AC**AGCA AACGTCTGTT G**G**TGAA**T**AAT TTTGATGAAC TGCTC**T**ATTT AATAAACTGT

wMel CATA**AC**AGCA AACGTCTGTT G**G**TGAA**T**AAT TTTGATGAAC TGCTC**T**ATTT AATAAACTGT

wHa CATA**AC**AGCA AACGTCTGTT G**G**TGAA**T**AAT TTTGATGAAC TGCTC**T**ATTT AATAAACTGT

wRi CATA**AC**AGCA AACGTCTGTT G**G**TGAA**T**AAT TTTGATGAAC TGCTCCATTT AATAAACTGT

wVulC CATA**AC**AGCA AACGTCTGTT G**G**TGAA**T**AAT TTTGATGAAC TGCTC**T**ATTT AATAAACTGT

**wStr** CATA**AC**AGCA AACGTCTGTT G**G**T**A**AA**T**AAT TTTGATGAAC TGCTC**T**ATTT AATAAACTGT

wTai CATAGTAGCA AACGTCTGTT G**G**T**A**AACAAT TTTGATGAAC TGCTCCATTT AATA**G**ACTGT

wPip CATAGTAGCA AACGTCTGTT GAT**A**AGCAAT TTTGATGAAC TGCTCCATTT AATAAACTGT

wVitB CATAGTAGCA AACGTCTGTT GAT**A**AGCAAT TTTGATGAAC TGCTCCATTT AATAAACTGT

wBm CATAGTAGCA AACG**CT**T**A**TT GAT**A**AACAAT TTTGATGAAC T**AT**T**T**CATTT A**G**TAAACTGT

**** **** **** * ** * * * *** ********** * * **** * ********

241 300

T I D E C A I

wKue TCAAAGGAAG AT**T**GCATAAA AGAGTTGCAA TGCTCAAAGA CAATAGATGA ATGTGCTATT

wMel TCAAAGGAAG AT**T**GCATAAA AGAGTTGCAA TGCTCAAAGA CAATAGATGA ATGTGCTATT

wHa TCAAAGGAAG AT**T**GCATAAA AGAGTTGCAA TGCTCAAAGA CAATAGATGA ATGTGCTATT

wRi TCAAAGGAAG AT**T**GCATAAA AGAGTTGCAA TGCTCAAAGA CAATAGATGA ATGTGCTATT

wVulC TCAAAGGAAG AT**T**GCATAAA AGAGTTGCAA TGCTCAAAGA CAATAGATGA ATGTGCTATT

**wStr** TCAAAGGAAG AT**T**GCATGAA AGAGTTGCAA TGCTCAAAGA CAATAGATGA ATGTGCTATT

wTai TCAAAGGAAG ATCACATAAA AGAATTGCAA TGCTCAAAGA CAATAGATGA ATATGCTATT

wPip TCAAAGGAAG **G**T**A**GCATAAA AGAGTTGCAA TGCTCAAAGA CAATAGATGC GTATGCTGTT

wVitB TCAAAGGAAG AT**T**G**G**ATAAA AGAGTTGCAA TGCTCAAAGA CAATAGATGC GTATGCTGTT

wBm TCAAA**A**GAAG ATCA**T**A**C**GAA **G**GAATT**A**CAA **C**G**T**TCAAAG**G** CAATAGATGA ATG**C**GCTATT

***** **** * * ** ** ** *** * ****** ********* * *** **

301 360

A L L K

wKue GCCTTGCTTA AGTTCTCAGA ATTATTGCCA TACGCGTTAG TGGCTGATAT GACTTTTGAG

wMel GCCTTGCTTA AGTTCTCAGA ATTATTGCCA TACGCGTTAG TGGCTGATAT GACTTTTGAG

wHa GCCTTGCTTA AGTTCTCAGA ATTATTGCCA TACGCGTTAG TGGCTGATAT GACTTTTGAG

wRi GCCTTGCTTA AGTTCTCAGA ATTATTGCCA TACGCGTTAG T**A**GCTGATAT GACTTTTGAG

wVulC GCCTTGCTTA AATTCTCAGA ATTATTGCCA TACGCATTAG TGGCTGATAT GACTTTTGAG

**wStr** GCCTTGCTTA AGTTCTCAGA ATTATTGCCA TACGCGTTAG TGGCTGATAT GACTTTTGAG

wTai GCCTTGCTTA AATTCTCAGA ATTATTGCCA TACGCATTAG TGGCTGATAT GAC**G**TTTGAG

wPip GCCTTGCTTA AGTTCTCAGA ATTATTGCCA TACGCGTTAG TGGCTGATAT GACTTTTGAG

wVitB GCCTTGCTTA AGTTCTCAGA ATTATTGCCA TACGCGTTAG TGGCTGATAT GACTTTTGAG

wBm **A**C**T**TTGCT**A**A AAT**CT**TCAGA ATT**G**TTGCCA TACGCATTAG TGG**T**TGA**CG**T GA**AC**TT**CA**A**A**

* ***** * * * ***** *** ****** ***** **** * * *** * ** ** *

361 420

wKue AATAA**C**CATG AAATGCGAAA TTGGTG**C**GA**G A**AAAATGAC**G** TTATTGCACT **GG**A**C**ACGT**C**A

wMel AATAA**C**CATG AAATGCGAAA TTGGTG**C**GA**G A**AAAATGAC**G** TTATTGCACT **GG**A**C**ACGT**C**A

wHa AATAA**C**CATG AAATGCGAAA TTGGTG**C**GA**G** **A**AAAATGAC**G** TTATTGCACT **GG**A**C**ACGT**C**A

wRi AATAA**G**TATG AAATGCGAAA TTGGTGTGAA GAAAATGACA TTATTGCACT A**G**ATACGTTA

wVulC AATAA**C**CATG AAATGC**A**AAA TTGGTG**C**GA**G A**AAAATGAC**G** TTATTGCACT **GG**A**C**ACGT**C**A

**wStr** AATAA**C**CATG AAATGCGAAA TTGGTG**C**GA**G A**AAAATGAC**G** TTATTGCACT **GG**A**C**A**A**GT**C**A

wTai AATAAACATG AAATGCGAAA TTGGTGTGAA GAAAATGACA TTATTGCACT AAATACGTTA

wPip AATAAACATG AAATGCGAAA TTGGTGTGAA GAAAATGACA TTATTGCACT AAATACGTTA

wVitB AATAAACATG AAATGCGAAA TTGGTGTGAA GAAAATGACA TTATTGCACT AAATACGTTA

wBm **G**AT**G**AA**T**ATG AAATGCGA**GG C**TGGTGTGAA **A**AAA**G**TGA**TG** T**A**ATTGCACT A**G**A**CGT**G**C**TA

** * *** ****** * ***** ** *** *** * ******** * * *

421 > **RibA GTP cyclohydrolase II** <480

wKue **T**T**CA**T**A**AAT**A** ATTTTCAA**G**A AAATCA**GGA**T GT**A**TATGAAG TGTGCAAAAC ATCATTATTT

wMel **T**T**CA**T**A**AAT**A** ATTTTCAA**G**A AAATCA**GGA**T GT**A**TATGAAG TGTGCAAAAC ATCATTATTT

wHa **T**T**CA**T**A**AAT**A** ATTTTCAA**G**A AAATCA**GGA**T GT**A**TATGAAG TGTGCAAAAC ATCATTATTT

wRi CT**C**GT**A**AATG ATTTTCAACA AAATCA**G**AGT GTGTATGAAG TGTGCAAAAC ATCATTATTT

wVulC **T**T**CA**T**A**AAT**A** ATTTTCAA**G**A AAATCA**GGA**T GT**A**TATGAAG TGTGCAAAAC ATCATTATTT

**wStr** **T**T**CA**T**A**AAT**A** ATTTTCAA**G**A AAATCA**GGA**T GT**A**TATGAAG TGTGCAAAAC ATCATTATTT

wTai CTTGTGAATG ATTTTCAAC**G** AAATCATAGT GTGTATGAAG TGTGCAAAAC ATCATTATTT

wPip CTTGTGAATG ATTTTCAACA AAATCATAGT GTGTATGAAG TGTGCAAAAC ATCATTATTT

wVitB CTTGTGAATG ATTTTCAACA AAATCATAGT GTGTATGAAG TGTGCAAAAC ATCATTATTT

wBm **T**TT**A**T**A**AAT**A** A**C**TTTCAACA AAATCA**GGA**T **A**T**A**TATGAAG T**A**TGCAAAAC A**C**CATTATTT

* * *** * ****** ****** * * ******* * ******** * ********

481 > **RibA GTP cyclohydrolase II** **domain** <540

wKue TTAAAACAGA CTCAAGAAGT AAATATCATA TCTTATAGAA CCGAAAGTGG TGGAAGAGAA

wMel TTAAAACAGA CTCAAGAAGT AAATATCATA TCTTATAGAA CCGAAAGTGG TGGAAGAGAA

wHa TTAAAACAGA CTCAAGAAGT AAATATCATA TCTTATAGAA CCGAAAGTGG TGGAAGAGAA

wRi TTAAAACAGA CTCAAGAAGT A**G**ATATCATA TCTTATAGAA CCGAAAGTGG **C**GGAAGAGAA

wVulC TTAAAACAGA CTCAAGAAGT A**G**ATATCATA TCTTATAGAA CCGAAAGTGG **C**GGAAGAGAA

**wStr** TTAAAGCAGA CTCAAGAAGT A**G**ATATCATA TCTTATAGAA CC**A**AAAGTGG **C**GGAAGAGAA

wTai TTAAAGCAGA CTCAAGAAGT A**G**ATATCATA TCTTATAGAA CC**A**AAAGTGG **C**GGAAGAGAA

wPip TTAAAGCAGA CTCAAGAAGT A**G**ATATCATA TCTTATAGAA CC**A**AAAGTGG **C**GGAAGAGAA

wVitB TTAAAACAGA CTCAAGAAGT A**G**ATATCATA TCTTATAGAA CC**A**AAAGTGG **C**GGAAGAGAA

wBm TTAAAACAGA CTCAA**A**AAGT AAA**C**ATCATA TCTTATAG**G**A CC**TGT**A**A**TGG **CA**GAA**A**AGAA

***** **** ***** **** * * ****** ******** * ** * *** *** ****

540 > **RibA GTP cyclohydrolase II** **domain** <600

wKue CATCATGCAA TTATCATTGG CAATCCAGAT AAAGATGACG AACCATTAGT GAGAATTCAT

wMel CATCATGCAA TTATCATTGG CAATCCAGAT AAAGATGACG AACCATTAGT GAGAATTCAT

wHa CATCATGCAA TTATCATTGG CAATCCAGAT AAAGATGACG AACCATTAGT GAGAATTCAT

wRi CATCATGCAA TTATCATTGG CAATCCAGAT AAAGATGACG AACCATTAGT AAGAATTCAT

wVulC CATCATGCAA TTATCATTGG CAATCCAGAT AAAGATGACG AACCATTAGT GAGAATTCAT

**wStr** CAT**T**ATGCAA TTATCATTGG CAATCCAGAT AAAGAT**A**A**T**G A**G**CCATTAGT GAGAATTCAT

wTai CAT**T**ATGCAA TTATCATTGG CAATCCAGAT AAAGAT**A**A**T**G A**G**CCATTAGT GAGAATTCAT

wPip CAT**T**ATGCAA TTATCATTGG CAATCCAGAT AAAGAT**A**A**T**G A**G**CCATTAGT GAGAATTCAT

wVitB CAT**T**ATGCAA TTATCATTGG CAATCCAGAT AAAGAT**A**A**T**G A**G**CCATTAGT GAGAATTCAT

wBm CAT**T**A**C**GCAA TTAT**T**ATTGG CAATCCAG**G**T AAA**A**AT**AGT**G AACCATTAGT **A**AGA**G**TTCAT

*** * **** **** ***** ******** * *** ** * * ******** *** *****

601 > **RibA GTP cyclohydrolase II** **domain** <660

wKue TCTTCGTGCT ATACGGGTGA CTTGTTAGAT AGCTTGTCAT GCGATTGCAG AAGTCAGTTA

wMel TCTTCGTGCT ATACGGGTGA CTTGTTAGAT AGCTTGTCAT GCGATTGCAG AAGTCAGTTA

wHa TCTTCGTGCT ATACGGGTGA CTTGTTAGAT AGCTTATCAT GCGATTGCAG AAGTCAGTTA

wRi TCTTCGTGCT ATACGGGTGA CTTGTTAGAT AGCTTATCAT GCGATTGCAG AAGTCAGTTA

wVulC TCTTCGTGCT ATACGGGTGA CTTGTTAGAT AGCTTATCAT GCGATTG**T**AG AAGTCAGTTA

**wStr** TCTTC**A**TGCT ATAC**A**GG**A**GA CTT**A**TTAGA**C** AGCTTATCAT G**T**GATTGCAG AAG**C**CAGT**CG**

wTai TCTTC**A**TGCT ATAC**A**GG**A**GA CTT**A**TTAGA**C** AGCTTATCAT G**T**GATTGCAG AAG**C**CAGTTA

wPip TCT**G**C**A**TGCT ATAC**A**GG**A**GA CTT**A**TTAGA**C** AGCTTATCAT G**T**GATTGCAG AAG**C**CAGTTA

wVitB TCTTC**A**TGCT ATAC**A**GG**A**GA CTT**A**TTAGA**C** AGCTTATCAT G**T**GATTGCAG AAG**C**CAGTTA

wBm TCTTC**A**TGCT ATAC**A**GG**C**GA CTT**A**TTAGAT AGCTTATCAT GCGATTGCAG AAGTCAGTTA

*** * **** **** ** ** *** ***** ***** **** * ***** ** *** ****

661 > **RibA GTP cyclohydrolase II** **domain** <720

wKue CATCAAGCAA TTCAAATGAT AGC**T**GACTCT GGAAGTGGTA TTATATTGTA TTTGATGCAA

wMel CATCAAGCAA TTCAAATGAT AGC**T**GACTCT GGAAGTGGTA TTATATTGTA TTTGATGCAA

wHa CATCAAGCAA TTCAAATGAT AGC**T**GACT**T**T GGAAGTGGTA TTATATTGTA TTTGATGCAG

wRi CATCAAGCAA TTCAAATGAT AGC**T**GACT**T**T GGAAGTGGTA TTATATTGTA TTTGATGCAG

wVulC CATCAAGCAA TTCAAATGAT AGC**T**GACT**T**T GGAAGTGGTA TTATATTGTA TTTGATGCAA

**wStr** CATCAAGC**G**A TTCAAAT**A**AT **GA**C**T**GACT**T**T GG**G**A**A**TGG**C**A TTATATT**A**TA **C**TTGATGCAA

wTai CATCAAGC**G**A TTCAAAT**A**AT **GA**CCGACT**T**T GG**G**A**A**TGG**C**A TTATATT**A**TA **C**TTGATGCAA

wPip CATCAAGC**G**A TTCAAAT**A**AT **GA**CCGACT**T**T GG**G**A**A**TGG**C**A TTATATT**A**TA **C**TTGATGCAA

wVitB CATCAAGC**G**A TTCAAAT**A**AT **GA**CCGACT**T**T GG**G**A**A**TGG**C**A TTATATT**A**TA **C**TTGATGCAA

wBm CATCAAGCAA TTCAAAT**A**AT **GA**C**T**GACTCT GG**G**A**A**TGGTA TTATA**C**T**A**TA **CC**TGATGCAA

******** * ******* ** * **** * ** * *** * ***** * ** *******

721 > **RibA GTP cyclohydrolase II** **domain** <780

wKue GATGGAAGAG GCATTGGTTT AACTAATAAG TTAAGAGCGT ACAGTATGCA AAGAGGACAT

wMel GATGGAAGAG GCATTGGTTT AACTAATAAG TTAAGAGCGT ACAGTATGCA AAGAGGACAT

wHa GATGGAAGAG GTATTGGTTT AACTAATAAG TTAAGAGC**A**T ACAGTATGCA AAGAGAACAT

wRi GATGGAAGAG GTATTGGTTT AACTAATAAG TTAAGAGC**A**T ACAGTGTGCA AAGAGAACAT

wVulC GATGGAAGAG GCATTGGTTT A**G**CTAATAAG TTAAGAGCGT ACAGTATGCA AAGA**A**GACAT

**wStr** GATGG**G**AGAG GCATTGGTTT AACTAATAAG TTAAGAGC**A**T ACAGTATGCA AAGA**A**AA**T**AT

wTai GATGG**G**AGAG GCATTGGTTT AACTAATAAG TTAAGAGC**A**T ACAGTATGCA AAGA**A**AA**T**AT

wPip GATGG**G**AGAG GCATTGGTTT AACTAATAAG TTAAGAGC**A**T ACAGTATGCA AAGA**A**AA**T**AT

wVitB GATGG**G**AGAG GCATTGGTTT AACTAATAAG TTAAGAGC**A**T ACAGTATGCA AAGA**A**AA**T**AT

wBm GATGG**T**AGAG GCATTGGTTT AACTAA**C**AAG TTAAG**G**GC**A**T A**TGA**TATGCA AAGA**A**AA**T**AT

***** **** * ******** * **** *** ***** ** * * ****** **** * **

781 > **RibA GTP cyclohydrolase II** **domain** <840

**V** L G F E D D E R

wKue AATCTTGATA CTGTTGATGC AAATAGAATA TTGGGTTTTG AAGATGATGA AAGGAGTTTT

wMel AATCTTGATA CTGTTGATGC AAATAGAATA TTGGGTTTTG AAGATGATGA AAGGAGTTTT

wHa AACCTTGATA CTGTTGATGC AAATAGAATA TTGGGTTTTG AAGATGATGA AAGGAGTTTT

wRi AACCTTGATA CTGTTGATGC AAATAGAATA TTGGGTTTTG AAGATGATGA AAGGAGTTTT

wVulC AATCTTGATA CTGTTGATGC AAATAGA**G**TA TTGGGTTTTG AAGATGATGA AAG**A**AG**C**TTT

**wStr** AATCTTGATA CTGTTGATGC AAATAGA**G**TA TTGGGTTTTG AAGATGATGA AAGGAG**C**TTT

wTai AATCTTGATA CTGTTGATGC AAATAGA**G**TA TTGGGTTTTG AAGATGATGA AAGGAG**C**TTT

wPip AATCTTGATA CTGTTGATGC AAATAGA**G**TA TTGGGTTTTG AAGATGATGA AAGGAG**C**TTT

wVitB AATCTTGATA CTGTTGATGC AAATAGA**G**TA TTGGGTTTTG AAGATGATGA AAGGAG**C**TTT

wBm AACCTTGATA CTGT**G**GATGC AAATAGAATA TTGGGTTTTG AAGATGATGA **G**AGGAGTTT**C**

** ******* **** ***** ******* ** ********** ********** ** ** **

841 > **RibA GTP cyclohydrolase II** **domain** <900

wKue GCTGTTGCAG CTAAAATGCT TAAGAAATTG AACATTAACA AAATCCAATT ACTTACAAAC

wMel GCTGTTGCAG CTAAAATGCT TAAGAAATTG AACATTAACA AAATCCAATT ACTTACAAAC

wHa GTTGTTGCAG CTAAAATGCT TAAGAAATTG AACATTAACA AAATCCAATT ACTTACAAAC

wRi GTTGTTGCAG CTAAAATGCT TAAGAAATTG AACATTAACA AAATCCAATT ACTTACAAAC

wVulC GCTGTTGCAG **T**TGAAAT**A**CT CAAGAAATTA GACATCAAAA A**G**ATCCAATT ACTTACAAAT

**wStr** GCTGTTGCAG CTAAAAT**A**CT TAAGAAATTG AACATTAACA AAAT**A**CAATT ACTTACAAAC

wTai GCTGTTGCAG CTAAAAT**A**CT TAAGAAATTG AACATTAACA AAAT**A**CAATT ACTTACAAAC

wPip GCTGTTGCAG CTAAAAT**A**CT TAAGAAATTG AACATTAACA AAAT**A**CAATT ACTTACAAAC

wVitB GCTGTTGCAG CTAAAAT**A**CT TAAGAAATTG AACATTAACA AAAT**A**CAATT ACTTA**A**AAAC

wBm GCTGTTGCAG CTGAAATGCT CAAGAAATTA GGCATCAAAA AAATCCA**GC**T ACTTACAAAT

********** * **** ** ******** *** ** * * ** ** * ***** ***

901 > **RibA GTP cyclohydrolase II** **domain** <960

wKue AATGATAGGA AATTGTCAGA ATTGGAAAGT AGTGGTATAG G**A**GTTACAAA GTGTCTACCA

wMel AATGATAGGA AATTGTCAGA ATTGGAAAGT AGTGGTATAG G**A**GTTACAAA GTGTCTACCA

wHa AATGATAGGA AATTGTCAGA ATTGGAAAGT AGTGGTATAG AGGTTACAAA GTGTCTACCA

wRi AATGATAGGA AATTGTCAGA ATTGGAAAGT AGTGGTATAG AGGTTACAAA GTGTCTACCA

wVulC AA**C**G**G**TAGGA A**G**TT**A**TC**G**GA **G**TTG**A**AAA**AC** A**A**TGGTATAG AGGTTACAAA GTGTCTACCA

**wStr** AATG**G**TAG**A**A A**G**TT**A**TCAGA **G**TTG**A**AAA**A**T A**AC**GGTATAG A**A**GTTACAAA GTGT**G**TACCA

wTai AATG**G**TAG**A**A A**G**TT**A**TCAGA **G**TTG**A**AAA**A**T A**AC**GGTATAG A**A**GTTACAAA GTGT**G**TACCA

wPip AATG**G**TAG**A**A A**G**TT**A**TCAGA **G**TTG**A**AAA**A**T A**AC**GGTATAG A**A**GTTACAAA GTGT**G**TACCA

wVitB AATG**G**TAG**A**A A**G**TT**A**TCAGA **G**TTG**A**AAA**A**T A**AC**GGTATAG A**A**GTTACAAA GTGT**G**TACCA

wBm AATG**G**TAG**A**A A**GC**T**A**TCAGA **G**TTG**A**AAA**A**T A**A**TGGTATAG AGGT**C**ACAAG **A**TGTCTACCA

** * *** * * * ** ** *** *** * ******* ** **** *** *****

961 > **RibA GTP cyclohydrolase II** **domain** <1020

wKue CTTATTGTGG AACGTAATAA ATATAACGAT TCATATATGG AGACAAAGTT TGGTAAATTA

wMel CTTATTGTGG AACGTAATAA ATATAACGAT TCATATATGG AGACAAAGTT TGGTAAATTA

wHa CTTATTGTGG AACGTAATAA ATATAACGAT TCATATATGG AGACAAAGTT TGGTAAGCTA

wRi CTTATTGTGG AACGTAATAA ATATAACGAT TCATATATGG AGACAAAGTT TGGTAAGCTA

wVulC CTTATT**A**TGG AACGTAAT**G**A ATATAA**T**GAT TCATATATGG A**A**ACAAA**A**TT TGGTAGGCTA

**wStr** CTTATT**A**TGG AACG**C**AAT**G**A ATATAA**T**GAT TCATATATGG A**A**ACAAA**A**TT TGG**C**AAGTTA

wTai CTTATT**A**TGG AACG**C**AAT**G**A ATATAA**T**GAT TCATATATGG A**A**ACAAA**A**TT TG**AC**AAGTTA

wPip CTTATT**A**TGG AACG**C**AAT**G**A ATATAA**TC**AT TCATATATGG A**A**ACAAA**A**TT TGG**C**AAGTTA

wVitB CTTATT**A**TGG AACG**C**AAT**G**A ATATAA**T**GAT TCATATATGG A**A**ACAAA**A**TT TGG**C**AAGTTA

wBm CTTATT**A**TGG AACGTAA**C**AA **G**TATAACGAT TC**G**TATAT**A**G A**A**ACAAA**A**TT T**A**G**C**AGGTTA

****** *** **** ** * ***** ** ** ***** * * ***** ** * * **

1021 > **Rib A domain** <1044

wKue GGCCATAGAT TAAGAGTTTT TTAG

wMel GGCCATAGAT TAAGAGTTTT TTAG

wHa GGCCATAAGT TAAGAGTTTT TTAG

wRi GGCCATAAGT TAAGAGTTTT TTAG

wVulC GGCCAT**G**GAT TAAG**G**GTTTT TTAG

**wStr** GGCCAT**G**GAT TAAG**G**GTTT**A** TTAG

wTai GGCCAT**G**GAT TAAG**G**GTTT**A** TTAG

wPip GGCCAT**G**GAT TAAG**G**GTTT**A** TTAG

wVitB G**A**CCAT**G**GAT TAAG**G**GTTT**A** TTAG

wBm GG**T**CA**C**AGAT TAAGA**AC**TTT TTAG

* ** * **** ** ****

**Figure S1.** Nucleotide alignment of *rib*A homologs from *^B^w*Str and WOL-A, B- and D-strains at left in red, blue and black font, respectively. Nucleotides encoding the dihydroxybutanone phosphate synthase and GTP cyclohydrolase II domains are indicated above the alignment within > < symbols. Asterisks below alignment indicate universally conserved nucleotides. Unique nucleotides are in green font. Nucleotides conserved in ^B^*w*Str and a majority of B-strains are in dark blue bold font, while those in dark red bold font are conserved with a majority of A-strains. Nucleotides conserved in two to four strains are in light blue, orange or orange bold font. Nucleotides highlighted in gray and cyan indicate the MS-detected ^A^*w*Mel and ^B^*w*Pip 95% confidence peptides shown in Fig. 1, with amino acids indicated at top. Underlined nucleotides correspond to the CAARTARY repeat. See Tables 2 and S2 for host associations and Genbank Accessions.
