# Supplementary material for: Mosaic composition of ribA and wspB genes flanking the virB8-D4 operon in the Wolbachia supergroup B-strain, wStr
Source: Arch Microbiol. 2015 Sep 23;198:53–69. doi: 10.1007/s00203-015-1154-8 (PMC4705124; doi:10.1007/s00203-015-1154-8)
Supplement: Supplementary file 5 — Nucleotide sequence alignment of wspB homologs from B wStr and WOL-A and B-strains as indicated by red and blue font at left. Nucleotides conserved between B wStr and a majority of A-strains are in red font and residues conserved with a majority of B-strains are in blue font. Asterisks below alignment indicate universally conserved nucleotides and double underlines above the alignment indicate three hypervariable regions (HVRs). Unique nucleotides are in green font and residues conserved between two to four strains are in light blue, orange or bold orange font. The greater than less than symbols below alignment indicate a transposon insertion in the wspB pseudogene of B wPip, which is aligned as three discontinuous sequence blocks corresponding to nucleotides 1334165 - 1334594; 1335958 - 1336167; 1336271 – 1336326 from Accession NC_010981.1. The three CAARTARY repeats are underlined (nucleotides 365–379 and 457–463). Highlighted residues correspond to 95% confidence peptides detected by LC–MS/MS (amino acids indicated at top; lower case indicates additional matched peptides not unique to WspB) that were conserved in most strains (gray), conserved in B-strains (cyan), conserved in B wStr and A wCobU4-2 (yellow), or unique to B wStr (olive). See Tables 2 and S2 for host associations and Genbank Accessions. (DOCX 271 kb) [file 203_2015_1154_MOESM5_ESM.docx]

**Figure S2.** Nucleotide sequence alignment of *wsp*B from ^B^*w*Str

1 60

wAtab3 ATGATTAGTA AAAAAACATT AGCGGTTACA GCATTTGCTT TATTGTTGTC ACAACAATCT

wKue ATGATTAGTA AAAAAACATT AGCGGTTACA GCATTTGCTT TATTGTTGTC ACAACAATCT

wMel ATGATTAGTA AAAAAACATT AGCGGTTACA GCATTTGCTT TATTGTTGTC ACAACAATCT

wRi ATGATTAGTA AAAAAACATT AGCGGTTACA GCATTTGCTT TATTGTTGTC ACAACAATCT

wAna ATGATTAGTA AAAAAACATT AGCGGTTACA GC**GC**TTGCTT TG**C**TGTTGTC ACAACAATCT

wCobU5-2---------- ---------- ---------- ---------- ---------- ----------

wCobU4-2---------- ---------- ---------- ---------- ---------- ----------

**wStr** ATGATGAGTA AAAAAACATT AGC**A**GTTACA GCA**C**TTGCTT TATTGTTGTC ACAACAATCT

wVitB ATGATGAGTA AAAAAACATT AGC**A**GTTACA GCA**C**TTGCTT TATTGTTGTC ACAACAATCT

wMet ---------- ---------- ---------- ---------- ---------- ----------

wNo ATGAGTAAAA AAACATTAGC AGTTACAGCA CTTGCTTTAT TGTTGTC--- ACAACAATCT

wPip ATGAGTAAAA AAACATTAGC AGTTACAGCA CTTGCTTTAT TGTTGTC--- ---ACAATCT

**** * * *** * * ** ** * * * *** *** **********

61 120

wAtab3 TTTGCAAGTG AAACAGAAGG GTTTTACTTT GGTAGTGGGT ATTACGGTCA ATATTT**A**AA**T**

wKue TTTGCAAGTG AAACAGAAGG GTTTTACTTT GGTAGTGGGT ATTACGGTCA ATATTT**A**AA**T**

wMel TTTGCAAGTG AAACAGAAGG GTTTTACTTT GGTAGTGGGT ATTACGGTCA ATATTT**A**AA**T**

wRi TTTGCAAGTG AAACAGAAGG GTTTTACTTT GGTAGTGGGT ATTACGGTCA ATATTT**A**AA**T**

wAna TTTGC**G**AGTG AAACAGAAGG GTTTTACTTT GGTAGTGGGT ATTACGGTCA ATATTT**A**AA**T**

wCobU5-2TTTGCAAGTG AAACAGAAGG GTTTTACTTT GG**C**AGTGGGT ATTACGGTCA AT**TA**C**ACGGG**

wCobU4-2TTTGCAAGTG AAACAGAAGG **A**TTTTACTTT GGT**G**GTGG**A**T ATTACGGTCA ATATTTGAAC

**wStr** TTTGCAAGTG AAACAGAAGG **A**TTTTACTTT GGT**G**GTGG**A**T ATTACGGTCA ATATCT**A**AA**T**

wVitB TTTGCAAGTG AAAC**G**GAAGG **A**TTTTACTTT GGT**G**GTGG**A**T ATTACGGTCA ATATTTGAAC

wMet TTTGCAAGTG AAACAGAAGG **A**TTTTACTTT GGT**G**GTGG**A**T ATTACGGTCA ATATTTGAAC

wNo TTTGCAAGTG AAACAGAAGG **A**TTTTACTTT GGT**G**GTGG**A**T ATTACGGTCA ATATTTGAAC

wPip TTTGCAAGTG AAACAGAAGG **A**TTTTACTTT GGT**G**GTGG**A**T ATTACGGTCA ATATTTGAAC

***** **** **** ***** ********* ** **** * ********** **

121  **HVR1 180**

**D A T D D N R V**

wAtab3 **G**ACACAAGCG TACTAAAAAC ---------- -----AAGCA CTACAGGTAT CAAAAATTTG

wKue AACACAAGCG TACTAAAAAC ---------- -----AAGCA CTACAGGTAT CAAAAATTTG

wMel AACACAAGCG TACTAAAAAC ---------- -----AAGCA CTACAGGTAT CAAAAATTTG

wRi AACACAAGCG TACTAAAAAC ---------- -----AAGCA CTACAGGTAT CAAAAATTTG

wAna -**---**----**T**G **AGT**T**G**AAA**G**C **AAAAATTGGA** **G**A**TA**CT**G**C**TG** CTACAGCTA**C** CAA**T**AAT**G**T**A**

wCobU5-2**T**ACA**TGG**G**T**G **AGT**T**G**AAA**G**C **AAAAATTGGA** **G**A**TA**CT**G**C**TG** CTACAGCT**GC** CAA**T**AAT**G**T**A**

wCobU4-**2T**---**TGG**G**TA** **AGT**T**G**AAA**G**C **AAAAATTGGA** **GGCAA**A**GATG** CTACAG**A**T**GA** **T**AA**CC**AT**G**T**A**

**wStr** **T**---**TGG**G**TA** **AGT**T**G**AAA**G**C **AAAAATTGGA** **GGCAA**A**GATG** CTACAG**A**T**GA** **T**AA**CCG**T**G**T**A**

wVitB **T**---**TGG**G**TA** **AGT**T**G**AAA**G**C **AAAAATTGGA** **GGCAA**A**GATG** CTACAG**A**T**GA** **T**AA**CCG**T**G**T**A**

wMet **T**---**TGG**G**TA** **AGT**T**G**AAA**G**C **AAAAATTGGA** **GGCAA**A**GATG** CTACAG**A**T**GA** **T**AA**CCG**T**G**T**A**

wNo **T**---**TGG**GT**A** **AGT**T**G**AAA**G**C **AAAAATTGGA** **G**A**TAAGGATG** CTACAG**A**T**GA** **T**AA**T**A**G**G**G**T**A**

wPip **T**---**TTG**G**TA** **AGT**T**G**AAA**G**C **AAAAATTGGA** **AGTAA**A**GATG** CTACAG**A**T**GC** CAA**T**AAG**G**T**A**

*** * * *** * ********** * ****** * ** *

**181 HVR1 HVR1** 240

S I N D **I D** A Q **R** T E G Q **L I** S **K**

wAtab3 TCTATAAATG ACAGGGGTGC TCAGAACACA GAAGGTCAGT CACTAAGTGA GTATAAAGGA

wKue TCTATAAATG ACAGGGGTGC TCAGAACACA GAAGGTCAGT CACTAAGTGA GTATAAAGGA

wMel TCTATAAATG ACAGGGGTGC TCAGAACACA GAAGGTCAGT CACTAAGTGA GTATAAAGGA

wRi TCTATAAATG ACAGGGGTGC TCAGAACACA GAAGGTCAGT CACTAAGTGA GTATAAAGGA

wAna TCT**G**TAAATG ACAGGAGTGC TCAGAACACA GAAGGTCAGT CACTAAGT**A**A GTATAAAGGA

wCobU5-2TCT**G**TAAATG ACAGGAGTGC TCAGAA**T**ACA GAAGGTCA**A**T CACTAAGT**A**A GTATAAAGGA

wCobU4-2TCTATAAATG ACA**TA**G**AC**GC TCAGA**GA**ACA GAAGGTCAGT **T**A**A**TAAGT**A**A GTATAAAGGA

**wStr** TCTATAAATG ACA**TA**G**AC**GC TCAGA**GA**ACA GAAGGTCAGT **T**A**A**TAAGT**A**A GTATAAAGGA

wVitB TCTATAAATG ACA**TA**G**AC**GC TCAGA**GA**ACA GAAGGTCAGT **T**A**A**TAAGT**A**A GTATAAAGGA

wMet TCTATAAATG ACA**TA**G**AC**GC TCAGA**GA**ACA GAAGGTCAGT **T**A**A**TAAGT**A**A GTATAAAGGA

wNo TTTATAAATG ACAG**A**A**ACA**C T**G**AAA**GA**ACA GAA**CC**TCAGC **C**A**A**TAAGTGA GTATAAAGCA

wPip TCT**G**TAAATG ACAGGGGTGC TCAGA**GT**ACA GAAGGTCAGC **T**ACTAA**ACA**A GTAT**G**AAGGA

*** ****** *** * * *** *** *** *** * *** * **** *****

241 300

V **T** F G Y T G E L G N

wAtab3 GATTATAATC CACCTTTTGC TGCAAATGTG GCATTTGGTT ACACAGGAGA ATTGGGTAAC

wKue GATTATAATC CACCTTTTGC TGCAAATGTG GCATTTGGTT ACACAGGAGA ATTGGGTAAC

wMel GATTATAATC CACCTTTTGC TGCAAATGTG GCATTTGGTT ACACAGGGGA ATTGGGTAAC

wRi GATTATAATC CACCTTTTGC TGCAAATGTG GCATTTGGTT ACACAGGAGA ATTGGGTAAC

wAna GATTATAATC C**G**CCTTTTGC TGCAAATGT**A** GCATT**G**GGTT ATACAGGGGA ATTGAATGGC

wCobU5-2GATTATAATC CACCTTTTGC TGCAAATGTG GCACTTGGTT ATACAGGGGA ATTGAATGGC

wCobU4-2GATTATAACC CACCTTTTGC TGCAAATGT**A** **A**C**G**TTTGGTT ACACAGGAGA A**C**T**A**GG**C**AA**T**

**wStr**  GATTATAA**A**C CACCTTTTGC TGCAAATGT**A** **A**C**G**TTTGGTT ACACAGGAGA A**C**T**A**GG**C**AA**T**

wVitB GATTATAACC CACCTTTTGC TGCAAATGT**A** **A**C**G**TTTGGTT ACACAGGAGA A**C**T**A**GG**C**AA**T**

wMet GATTATAACC CACCTTTTGC TGCAAATGT**A** **A**C**G**TTTGGTT ACACAGGAGA A**C**T**A**GG**C**AA**T**

wNo GATTATAGTC CACCTTTTGC TGCAAATAT**A** GCATTTGGTT ACACAGGAGA ATTGGGTAA**T**

wPip GATTATAATC CACCTTTTGC TGCAAATGT**A** GCACTTGGTT ATACAGGGGA ATT**A**GGTAA**T**

******** * * ******** ********* * * **** * ***** ** * *

301 360

N S Y R A E L E G M Y S S V K V D N I G

wAtab3 AACAGCTATA GGGCTGAATT GGAAGGGATG TATTCTTCTG TAAAAGTGGA TAATATTGGT

wKue AACAGCTATA GGGCTGAATT GGAAGGGATG TATTCTTCTG TAAAAGTGGA TAATATTGGT

wMel AACAGCTATA GGGCTGAATT GGAAGGGATG TATTCTTCTG TAAAAGTGGA TAATATTGGT

wRi AACAGCTATA GGGCTGAATT GGAAGGGATG TATTCTTCTG TAAAAGTGGA TAATATTGGT

wAna AA**T**AGCTACA G**A**GCTGAATT GGAAGGAATG TATTCTTCTG TAAAAGTGGA TAATATTGGT

wCobU5-2AA**T**AGCTACA G**A**GCTGAATT GGAAGGAATG TATTCTTCTG TAAAAGTGGA TAATATTGGT

wCobU4-2AA**T**AGCTATA G**A**GCTGAATT GGAAGGGATG TA**C**TCTTCTG TAAAAGT**A**GA TAATATTGGT

**wStr**  AA**T**AGCTATA G**A**GCTGAATT GGAAGGGATG TA**C**TCTTCTG TAAAAGT**A**GA TAATATTGGT

wVitB AA**T**AGCTATA G**A**GCTGAATT GGAAGGGATG TA**C**TCTTCTG TAAAAGT**A**GA TAATATTGGT

wMet AA**T**AGCTATA G**A**GCTGAATT GGAAGGGATG TA**C**TCTTCTG TAAAAGT**A**GA TAATATTGGT

wNo AA**T**AGTTATA G**A**GC**C**GAATT AGAAGGAAT**A** TATTCTTCT**A** TAAAAGTG**A**A TAATATTGGT

wPip AA**T**AGCTATA G**A**GCTGAATT AGAAGGAATG TATTCTTC**A**G T**G**AAAGTGGA TAATATT**C**GT

** ***** * * ** ***** ***** *** ** ***** * ***** * ******* *

**361 HVR2 HVR2 420**

L **S** S **N** Q  **V** T V S Y L K **D V** G E **S** **A** N K

wAtab3 TTAA**CAAGTA G**C**CAAATAA**C TGTTTCATAC CTAAAGGAGA CTGGTGAGGA TCCTAATAAA

wKue TTAA**CAAGTA G**C**CAAATAA**C TGTTTCATAC CTAAAGGAGA CTGGTGAGGA TCCTAATAAA

wMel TTAA**CAAGTA G**C**CAAATAA**C TGTTTCATAC CTAAAGGAGA CTGGTGAGGA TCCT**G**ATAAA

wRi TTAA**CAAGTA G**C**CAAATAA**C TGTTTCATAC CTAAAGGAGA CTGGTGAGGA TCCTAATAAA

wAna TTAACAAGCG GCCAGATGAC AATATCCTAC ACAAAGGATA AAACTA**G**A-- ----------

wCobU5-2TTAGCAAGCA GCCAGATAAC AATATCCTAC ATAAAGGATA AAGCTAA**C-- ----------**

wCobU4-2TTA**TCAAGTA ATCAAGTAA**C TGTTTC**G**TAT CTAAA**A**GA**CG** **T**TGGTGAG**AG** T**G**CTAATAAA

**wStr** TTA**TCAAGTA ATCAAGTAA**C TGTTTC**G**TA**T** CTAAA**A**GA**CG** **T**TGGTGAG**AG** T**G**CTAATAAA

wVitB TTA**TCAAGTA ATCAAGTAA**C TGTTTC**G**TA**T** CTAAA**A**GA**CG** **T**TGGTGAG**AG** TACTAATAAA

wMet TTA**TCAAGTA ATCAAGTAA**C TGTTTC**G**TA**T** CTAAA**A**GA**CG** **T**TGGTGAG**AG** TACTAATAAA

wNo TTAGCAAACA **CT**CAGATGA**A** TATT**AAG**TA**T** G**A**AAA**A**GA**A**A ACA**A**TAAA-- ----------

wPip TTAACAAGCG GCCAGATGAC AATATC**G**TAC A**C**A**G**AAGG**CG** GCA**A**T**C**AA-- ----------

*** **** 🡹** * * * ** * * ** * * ** *****

**421 HVR2 HVR2** 480

**k** t y **m** y **k** **T** **V I N** H D Q **V** E N **A** S V M

wAtab3 GAAACTTATC TCTATAGTGC TGCAGTTAGT CATGACCAAA TTGAGAACAT ATCTGTAATG

wKue GAAACTTATC TCTATAGTGC TGCAGTTAGT CATGACCAAA TTGAGAACAT ATCTGTAATG

wMel GAAACTTATC TCTATAGTGC TGCAGTTAGT CATGACCAAA TTGAGAACAT ATCTGTAATG

wRi GAAACTTATC TCTATAGTGC TGCAGTTAGT CATGACCAAA TTGAGAACAT ATCCGTAATG

wAna CCAGAAGAG**A** GCTATGG**A**GC AA**T**AGTCA**A**T CATGACCAAA TTGAGAACAT ATCTGTAATG

wCobU5-2CCAGAAGAG**A** GATATGG**A**GC AA**T**AGTCA**A**T CATGACCA**G**A T**A**GAGAAC**GC** **G**TCT**T**TAATG

wCobU4-2**A**AAACTTAT**A** T**G**TATA**AAA**C TG**T**A**A**TTA**A**T CATGAC**CAAG TAG**A**A**AAC**GC** ATCTGTAATG

**wStr** **A**AAACTTAT**A** T**G**TATA**AAA**C TG**T**A**A**TTA**A**T CATGAC**CAAG TAG**A**A**AAC**GC** ATCTGTAATG

wVitB **A**AAACTTAT**A** T**G**TATA**AAA**C TG**T**A**A**TTA**A**T CATGAC**CAAG TAG**A**A**AAC**GC** ATCTGTAATG

wMet **A**AAACTTAT**A** T**G**TATA**AAA**C TG**T**A**A**TTA**A**T CATGAC**CAAG TAG**A**A**AAC**GC** ATCTGTAATG

wNo ---------- --TATGGTG**T** TAC**GA**TTA**A**T CATGGCAAAA TTGA**T**AACAT ATCTGTAATG

wPip ---------- TATATGGA**T**C AA**TTA**TT**C**A**C** CATGACAAAA T--------- ATCTGTAATG

* * *** * ****** * *>** *** < *** *****

481 540

A N V Y H **Y** W K S D **S** **L** S F S P Y V G V

wAtab3 GCAAATGTTT ATCATCATTG GAAAAGTGAC CGTTTCTCTT TTTCTCCTTA CGTTGGTATT

wKue GCAAATGTTT ATCATCATTG GAAAAGTGAC CGTTTCTCTT TTTCTCCTTA CGTTGGTATT

wMel GCAAATGTTT ATCATCATTG GAAAAGTGAC CGTTTCTCTT TTTCTCCTTA CGTTGGTATT

wRi GCAAATGTTT ATCATCATTG GAAAAGTGAC CGTTTCTCTT TTTCTCCTTA CGTTGGTATT

wAna GCAAATGTTT ATCATCATTG GAAAAGTGAC CGTTTCTCTT TTTCTCCTTA **T**GTTGGG**G**TT

wCobU5-2GCAAATGTTT ATCATCATTG GAAAAGTGAC CGTTTCTCTT TTTCTCCTTA CGTTGGTATT

wCobU4-2GCAAATGTTT ATCAT**T**ATTG GAAAAGTGA**T** **A**GTTTCTCTT TTTCTCCTTA **T**GTTGGG**G**TT

**wStr** GCAAATGTTT A**C**CAT**T**ATTG GAAAAGTGA**T** **A**GTTT**A**TCTT TTTCTCCTTA **T**GTTGG**A**GTT

wVitB GCAAATGTTT ATCAT**T**ATTG GAAAAGTGA**T** **A**GTTTTTCTT TTTCTCCTTA CGTTGGTATT

wMet GCAAATGTTT ATCAT**T**ATTG GAAAAGTGA**T** **A**GTTTTTCTT TTTCTCCTTA CGTTGGTATT

wNo GCAAATGTTT ATCATCATTG GAAAAATGA**T** **A**GTTTCTCTT TTTCTCCTTA CGTTGGTATT

wPip GCAAATGTTT ATCATCATTG GAAAAGTGA**G** **A**GTTTCTCTT TTTCTCCTTA **T**GTTGGTATT

********** * *** **** ********* **** **** ********** ***** **

541 600

G **V** G A T R **p** s i r p a g q

wAtab3 GGGAT**C**GGTG CAACAAGAAT GACGATGTTT GAAAAA**C**CGT CAATAAGACC **C**GCAGGTCAA

wKue GGGAT**C**GGTG CAACAAGAAT GACGATGTTT GAAAAA**C**CGT CAATAAGACC **C**GCAGGTCAA

wMel GGGAT**C**GGTG CAACAAGAAT GACGATGTTT GAAAAA**C**CGT CAATAAGACC **C**GCAGGTCAA

wRi GGGAT**C**GGTG CAACAAGAAT GACGATGTTT GAAAAA**C**CGT CAATAAGACC **C**GCAGGTCAA

wAna GG**AG**T**C**GGTG CAACAAGAAT GAC**A**ATGTTT GAAAAATCGT CAATAAGACC **C**GCAGGTCAA

wCobU5-2GG**AG**T**C**GGTG CAACAAGAAT GA**AA**ATGTTT GAAAAATCGT CAATAAGACC TGCAGGTCAA

wCobU4-2GG**AG**T**C**GGCG CAACAAGAAT GAC**A**ATGTTT GAAAAATCGT CAATAAGACC TGCAGGTCAA

**wStr** GG**AG**TCGGTG CAACAAGAAT GAC**A**ATGTTT GAAAAA**C**CGT CAATAAGACC **C**GCAGGTCAA

wVitB GG**AG**TTGGTG GAACAAGAAT GACGATGTTT GAAAAATCGT CAATAAGACC TGCAGGTCAA

wMet GG**AG**TTGGTG GAACAAGAAT GACGATGTTT GAAAAATCGT CAATAAGACC TGCAGGTCAA

wNo GG**AG**TTGGTG CAACAAGAAT GAC**A**ATGTTT GAAGAATCGT CAATAAGACC TGCAGGTCAA

wPip GG**AG**TTGGTG CAACAAGAAT GAC**A**ATGTTT GAAAAATCGT CAATAAGACC TGCAGGTCAA

** * **** ********* ** ****** ****** *** ********** *********

601 660

l k a g f d y r I N E D V N M H I G Y R

wAtab3 TTAAAAGCTG GCTTTGACTA TCGCATAAAC GAAGATGTAA ATATGCATAT CGGATATAGA

wKue TTAAAAGCTG GCTTTGACTA TCGCATAAAC GAAGATGTAA ATATGCATAT CGGATATAGA

wMel TTAAAAGCTG GCTTTGACTA TCGCATAAAC GAAGATGTAA ATATGCATAT CGGATATAGA

wRi T**C**AAAAGCTG GCTTTGACTA TCGCATAAAC GAAGATGTAA ATATGCATAT CGGATATAGA

wAna TTAAAAGCTG GCCTTGA**T**TA TCGCATAAAC GAAGATGTAA ATATGCATAT CGGATATAGA

wCobU5-2TT**G**AAAGCTG GCTTTGA**T**TA TCG**T**ATAAAC GAAGATGTAA ATAGGCA**C**AT CGGATATAGA

wCobU4-2TT**G**AAAGCTG GCTTTGA**T**TA TCG**T**ATAAAC GAAGATGTAA ATAGGCA**C**AT CGGAT**G**TAGA

**wStr** TT**G**AAAGCTG GCTTTGA**T**TA TCG**T**ATAAAC GAAGATGTAA ATATGCA**C**AT CGGATATAGA

wVitB TT**G**AAAGCTG GCTTTGA**T**TA TCG**T**ATAAAC GAAGATGTAA ATATGCA**C**AT CGGATATAGA

wMet TT**G**AAAGCTG GCTTTGA**T**TA TCG**T**ATAAAC GAAGATGTAA ATATGCA**C**AT CGGATATAGA

wNo TTAAAGGCTG GCTTTGA**T**TA TCA**T**ATAAA**T** GAAGATGTAA ATATGCATAT TGGATATAGA

wPip TTAAAGGCTG GCTTTGA**T**TA TCG**T**ATAAAC GAAGATGTAA ATATGCA**C**AT CGGATATAGA

* ******* ******* ** *** ***** ********** *** *** ** **********

661 **HVR3 HVR3 720**

G F G **V L** G S **N V D F E** A **E V** L G **E M K**

wAtab3 GGTTTTGGTG CTATTGGTAG CGATATTAAG CTTACAGCAA AAAGGTTAGG ACAAGTGGTA

wKue GGTTTTGGTG CTATTGGTAG CGATATTAAG CTTACAGCAA AAAGGTTAGG ACAAGTGGTA

wMel GGTTTTGGTG CTATTGGTAG CGATATTAAG CTTACAGCAA AAAGGTTAGG ACAAGTGGTA

wRi GGTTTTGGTG CTATTGGTAG CGATATTAAG CTTACAGCAA AAAGGTTAGG ACAAGTGGTA

wAna GGTTTTGGTG CTATTGGTAG C--------- ---AGTGA**G**T A**T**AAACTAGA **C**AC**C**TTA**AA**A

wCobU5-2GGTTTTGGTG **T**T**C**TTGGTAG **TA**AT**G**TT**G**A**T** **T**TT**GA**AGCA**G** A**GGT**GTTAGG A**G**AAATG**AA**A

wCobU4-2GGTTTTGGTG **T**T**C**TTGGTAG **TA**AT**G**TT**G**A**T** **T**TT**GA**AGCA**G** A**GGT**GTTAGG A**G**AA**A**TG**AA**A

**wStr** GGTTTTGGTG **T**T**C**TTGGTAG **TA**AT**G**TT**G**A**T** **T**TT**GA**AGCA**G** A**GGT**GT**A**AGG A**G**AA**A**TG**AA**A

wVitB GGTTTTGGTG **T**T**C**TTGGTAG **TA**AT**G**TT**G**A**T** **T**TT**GA**AGCA**G** A**GGT**GTTAGG A**G**AA**A**TG**AA**A

wMet GGTTTTGGTG **T**T**C**TTGGTAG **TA**AT**G**TT**G**A**T** **T**TT**GA**AGCA**G** A**GGT**GTTAGG A**G**AA**A**TG**AA**A

wNo GGTTTTGGTG **T**TATTGGTAG C--------- ---AGTGAAT A**C**AAAC**C**GGA AAC**T**TTA**AAG**

wPip GGTTTTGGTG **T**T**C**TTGGTAG ---------- ---------- ---------- ----------

********** * ******* ** ** * ** * ** * *

**721 HVR3**  **k i l e l n k** **HVR3 780**

wAtab3 GACGACCCTA ATAATGATAA AAA-AAAG-- ---------- ---------- -AAGCTTAAT

wKue GACGACCCTA ATAATGATAA AAA-AAAG-- ---------- ---------- -AAGCTTAAT

wMel GACGACCCTA ATAATGATAA AAA-AAAG-- ---------- ---------- -AAGCTTAAT

wRi GACGACCCTA ATAATGATAA AAA-AAAG-- ---------- ---------- -AAGCTTAAT

wAna **TGG**GATCC**C**A AC**C**ATGATAA **TGGC**AAAG**AT AAGCCAAAAG GTGGAATGGC** T**GA**AC**AA**A**CA**

wCobU5-2G**TAA**A**G**C**AAC** A**GGTAA**A**CCC** A**G**A**TGG**A**AAA** **AAGATATTAG** **AATTGAATAA** **A**AG**T**C**AA**AA**A**

wCobU4-2G**TAA**A**G**C**AAC** A**GGTAA**A**CCC** A**G**A**TGG**A**AAA AAGATATTAG AATTGAATAA A**AG**T**C**AA**AA**A**

**wStr** GC**AA**A**G**C**AAC** **CGGTAA**A**CCC** A**G**A**TGG**A**AAA AAGATATTAG AATTGAATAA** **A**AG**T**C**AA**AA**A**

wVitB GC**AA**A**G**C**AAC** A**GGTAA**A**CC**A A**G**A**TGG**A**AAA AAGATATTAG** **AATTGAATAA** **A**AA**T**C**AA**AA**A**

wMet GA**AA**A**G**C**AAC** A**GGCAA**A**CTC** A**G**A**TGG**A**AAA AAGATATTAG AATTGAATAA** **A**AA**T**C**AA**AA**A**

wNo **A**A**A**G**T**T**GAAT** **G**C**T**A**AA**A**C**AA AAA**A**AA**T**G-- -------------------**AA** TAAAC**AA**A**T**A

wPip ---------- ---------- ---------- ---------- ---------- ----------

* * * * * *** * ** ** * * *

**781 HVR3 HVR3** 840

wAtab3 CCTAGCTCAG GTAGCAAAGT AACTGAGGAA ATAAATATAG GTAATCAACT ATTTCACACA

wKue CCTAGCTCAG GTAGCAAAGT AACTGAGGAA ATAAATATAG GTAATCAACT ATTTCACACA

wMel CCTAGCTCAG GTAGCAAAGT AACTGAGGAA ATAAATATAG GTAATCAACT ATTTCACACA

wRi CCTAGCTCAG GTAGCAAAGT AACTGAGGAA ATAAATATAG GTAATCAACT ATTTCACACA

wAna ---------- --GGTGA**T**A**A** T**CAA**GT**ATCT** ACTACCATAC AGAA**CG**ACTT TTTTCACACA

wCobU5-2CC**ATCGGACC** **AG**A**AGTT**A**CA** **C**A**AA**GA**AAGT** AT**C**A**G**TATAG G**C**AATCAA**G**T **G**TTTCACACA

wCobU4-2CC**ATCGGACC AG**A**AGTT**A**CA C**A**AA**GA**AAGT** AT**C**A**G**TATAG G**C**AATCAA**G**T **G**TTTCACACA

**wStr** CC**ATCGGACC** **AG**A**AGTT**A**CA** **C**A**AA**GA**AAGT** AT**C**A**G**TATAG G**C**AATCAA**G**T **G**TTTCACACA

wVitB CC**GTCGGACC** **AG**A**AGTT**A**TA** **C**A**AA**GA**AAGT** AT**C**A**G**TATAG G**C**AATCAA**G**T **G**TTTCACACA

wMet CC**ATCTGACC** **AG**A**AGTT**A**CA** **C**A**AA**GA**AAGT** AT**C**A**G**TATAG G**C**AATCAA**G**T **G**TTTCACACA

wNo ---------- --GGTGA**G**A**A** TA**AA**GT**AA**CT **G**CT**G**CCATAC AGAAT**AG**CTT TTTTCACACA

wPip ---------- -------------------------------- ---------- -TTTCACACA

** * * * *** ** * *********

841 878

wAtab3 CACGGTATAG AGGCTGGTCT TACTTTCCAT TTTGCCAGCA AAGCTTAA

wKue CACGGTATAG AGGCTGGTCT TACTTTCCAT TTTGCCAGCA AAGCTTAA

wMel CACGGTATAG AGGCTGGTCT TACTTTCCAT TTTGCCAGCA AAGCTTAA

wRi CACGGTATAG AGGCTGGTCT TACTTTCCAT TTTGCCAGCA AAGCTTAA

wAna CACGG**C**ATAG AGGCTGGTCT TACTTTCCAC TTTGC**T**AGTA AAGCTTAA

wCobU5-2CACGGTATAG A**A**GCTGGCCT TACTTTCCAT TTTGCCAGCA AAGC**A**---

wCobU4-2CACGGTATAG A**A**GCTGGTCT TACTTTCCAT TTTGCCAGCA AAGCA---

**wStr** CACGTTATAG A**A**GCTGGTCT TACTTTTCAT TTTGCCAGTA AAGCTT**G**A

wVitB CACGGTATAG A**A**GCTGGTCT TACTTTTCAT TTTGCCAGCA AAGCTTAA

wMet CACGGTATAG A**A**GCTGGTCT TACTTTTCAT TTTGCCAGCA AAGC----

wNo CACGGTATAG A**A**GCTGGTCT TACTTTTCAT TTTGCCAGCA AAGCTTAA

wPip CACGGTATAG A**A**GCTGGTCT TACTTTTCAT TTTGCCAGCA AA**AGC**T**T**-

***** **** * ******** ********** ***** ** * ** * *

**Figure S2.** Nucleotide sequence alignment of *wsp*B homologs from ^B^*w*Str and WOL*-*A and B-strains as indicated by red and blue font at left. Nucleotides conserved between ^B^*w*Str and a majority of A-strains are in red font and residues conserved with a majority of B-strains are in blue font. Asterisks below alignment indicate universally conserved nucleotides and double underlines above the alignment indicate three hypervariable regions (HVRs). Unique nucleotides are in green font and residues conserved between two to four strains are in light blue, orange or bold orange font. The > < symbols below alignment indicate a transposon insertion in the *wsp*B pseudogene of ^B^*w*Pip, which is aligned as three discontinuous sequence blocks corresponding to nucleotides 1334165 - 1334594; 1335958 - 1336167; 1336271 – 1336326 from Accession NC_010981.1. The three CAARTARY repeats are underlined (nucleotides 365-379 and 457-463). Highlighted residues correspond to 95% confidence peptides detected by LC-MS/MS (amino acids indicated at top; lower case indicates additional matched peptides not unique to WspB) that were conserved in most strains (gray), conserved in B-strains (cyan), conserved in ^B^*w*Str and ^A^*w*CobU4-2 (yellow), or unique to ^B^*w*Str (olive). See Tables 2 and S2 for host associations and Genbank Accessions.
