# Supplementary material for: Mosaic composition of ribA and wspB genes flanking the virB8-D4 operon in the Wolbachia supergroup B-strain, wStr
Source: Arch Microbiol. 2015 Sep 23;198:53–69. doi: 10.1007/s00203-015-1154-8 (PMC4705124; doi:10.1007/s00203-015-1154-8)
Supplement: Supplementary file 6 — Nucleotide sequence alignment of ribB homologs from B wStr and WOL-A, B- and D-strains at left in red, blue and black font, respectively. Asterisks below the alignment indicate universally conserved nucleotides. Unique nucleotides are in green font. Nucleotides conserved in B wStr and a majority of B-strains are in dark blue bold font, while those in dark red bold font are conserved with a majority of A-strains. Nucleotides conserved in two to four strains are in light blue, orange or orange bold font. See Tables 2 and S2 for host associations and Genbank Accessions. (DOCX 250 kb) [file 203_2015_1154_MOESM6_ESM.docx]

Fig. S3. Nucleotide sequence alignment of *rib*B from *^B^w*Str

1 60

wAna GGTATTTCTT CTGTGGAAGA TGTATTAGAG GATGCTCGTT CCGGTAAATT ATTCATTTTA wHa GGTATTTCTT CTGTGGAAGA TGTATTAGAG GATGCTCGTT CCGGTAAATT ATTCATTTTA

wRi GGTATTTCTT CTGTGGAAGA TGTATTAGAG GATGCTCGTT CCGGTAAATT ATTCATTTTA

wPip GGTATTTCTT CTGTGGAAGA TGTATTGAGG GATGCT**A**GTT CTGGTAAATT ATTCATTTTA

wAu GGTATTTCTT CTGTGGAAGA TGTATTAGAG GATGCTCGTT CCGGTAAATT GTTCATTTTA

wMel GGTATTTCTT CTGTGGAAGA TGTATTAGAG GATGCTCGTT CCGGTAAATT GTTCATTTTA

**wStr** GGTATTTCTT CTGTGGAAGA TGTATTAGAG GATGCGCGTT CCGGTAAATT ATTTATTTTA

wNo **CT**CATTTCTT CTGTGGAAGA T**A**TATTAGAG GATGTGCGTT CCGGTAAATT ATTCATTTTA

wBm GGCATTTCTT CTGTGGAAGA TGT**G**TTAGA**A** GATGCTCGTT **T**TGGTAA**G**TT GTTTATTTTA

******* ********** * * ***** ****** *** ***** ** ** ******

61 120

wAna GTTGATGATG AAAATAGAGA AAATGAAGGC GATTTGATTG TCTTAGCTGA GAAAATAAAG

wHa GTTGATGATG AAAATAGAGA AAATGAAGGC GATTTGATTG TCTTAGCTGA GAAAATAAAG

wRi GTTGATGATG AAAATAGAGA AAATGAAGGC GATTTGATTG TCTTAGCTGA GAAAATAAAG

wPip GTTGACGATG AAAA**C**AGAGA GAATGAAGGT GATCTGATTG T**A**TTAGCTGA GAAA**T**TAGAG

wAu GTTGATGATG AAAGTAGAGA GAATGAAGGT GATTTGGTTG TCTTAGCTGA AAAAGTAAAA

wMel TTTA**G**TT**G**A**T** **G**A**T**GAAA**GT**A GA**GA**GAA**T**G**A** A**GG**T**G**A**T**TTG **G**TT**GTC**TT**AG** **CTG**AA**A**AA**GT**

**wStr** TTTA**T**TT**T**AG T**TG**ATAA**T**GA AAAT**AG**AG**AG** AAT**GA**AG**G**TG **A**TTT**G**GTTG**T** **TTT**AG**CT**GAA

wNo GTTGAT**A**ATG AAAATAGAGA GAATGAAGGT GATTTGGTTG TTTTAGCTGA AAA**G**GT**G**AAA

wBm GTTGATGATG A**G**AATAGAGA AAATGAAGGT GATCTGAT**C**G TCTTAGCTGA AAAA**C**TAAAG

** * * * * * * * *

121 180

wAna ---------C CGGAACATAT GGCTTTTATG GTTAGGTA**C**G GTAC**A**GG**G**GT TGT**T**T**GC**TTA

wHa ---------C CGGAACATAT GGCTTTTATG GTTAGGTA**C**G GTAC**A**GG**G**GT TGT**T**T**GC**TTA

wRi ---------C CGGAACATAT GGCTTTTATG GTTAGGTA**C**G GTAC**A**GG**G**GT TGT**T**T**GC**TTA

wPip ---------C CAGAACATGT GGCTTTTATG GTTAGATA**C**G GTAC**A**GG**G**AT TGT**T**T**GC**TTA

wAu ---------C CAGAACATAT GGCTTTTATG GTTAGATA**C**G GTACTGGTAT TGTATTTTTA

wMel -----**A**AAAC CAGAACATAT GGCTTTTATG GTTAGATA**C**G GTACTGGTAT TGTATTTTTA

**wStr** **AAGGTG**AAGC CAGAACATAT TGCTTTTATG GTTAGGTATG GCACTGGTAT TGTGTTTTTA

wNo ---------C CAGAACATAT TGCTTTTATG GTTAGGTATG GCACTGGTAT TGTGTTTTTA

wBm ---------C CAGA**G**CAT**G**T GGCTTTTATG GTTAGGTATG GTACTGGTAT TGTATTT**C**TA

* **** *** * ********* ******** * * ** ** * *** * **

181 240

wAna GC**A**ATGAC**T**A A**A**TTTCACAT GA**AA**AGG**T**TG GGCCTT**A**A**T**T T**C**ATG**G**AAA**A** GA**AA**AATAT**T**

wHa GC**A**ATGAC**T**A A**A**TTTCACAT GA**AA**AGG**T**TG GGCCTT**A**A**T**T T**C**ATG**G**AAA**A** GA**AA**AATAT**T**

wRi GC**A**ATGAC**T**A A**A**TTTCACAT GA**AA**AGG**T**TG GGCCTT**A**A**T**T T**C**ATG**G**AAA**A** GA**AA**AATAT**T**

wPip GC**A**ATGAC**T**A A**AG**TTCACAT GA**AA**AGG**T**TA GGCCTT**AGT**T T**C**ATG**G**AAA**A** **A**A**AA**AAT**G**T**T**

wAu GCTATGACAA AGCTTCATAT GAGTA**AA**CTA AATCTTGAGT TTATGAG**G**A**A** GAGCAAT**G**TA

wMel GCTATGACAA AGCTTCATAT GAGTA**AA**CTA **A**ATCTTGAGT TTATGAG**G**A**A** GAGCAAT**G**TA

**wStr** GCTATGACGA AGTTTTACAT GAATA**AA**CTT **A**GCCTTGAAT TTATGAA**G**AG **A**AGTAAT**G**TA

wNo GCTATGACAA AGTTTTACAT GAATA**AA**CTT **A**ACCTTGAAT TTATGAA**G**AG **A**AGTAAT**G**TA

wBm GCTATGACAA **G**GCTTCATAT G**G**GTAGGCTT GGTCTTGAGT TTATGAGAAG **A**AGTAATATA

** ***** * ** * ** * * * *** * * *** * * ***

241 300

wAna G**G**TGAAAA**T**C **A**TACC**G**CA**TT** TACT**A**CAT**CA** ATT**GATG**CA**C** **G**T**T**ATG**G**C**AT** T**AC**AA**C**CGGT

wHa G**G**TGAAAA**T**C **A**TACC**G**CA**TT** TACT**A**CAT**CA** ATT**GATG**CA**C** **G**T**T**ATG**G**C**AT** T**AC**AA**C**CGGT

wRi G**G**TGAAAA**T**C **A**TACC**G**CA**TT** TACT**A**CAT**CA** ATT**GATG**CA**C** **G**T**T**ATG**G**C**AT** T**AC**AA**C**CGGT

wPip G**G**TGAAAA**T**C **A**TACT**G**CT**TT** TACT**A**CAT**CA** ATT**GATG**CA**C** **G**T**T**ATG**GTAT** T**AC**T**ACGGG**T

wAu GATGAAAAGC TTACTCCTCA TACTGCATTT ACTAC**G**TCAA TTGATGCGCG TTATGGCATT

wMel GATGAAAAGC TTACTCCTCA TACTGCATTT ACTAC**G**TCAA TTGATGCGCG TTATGGCATT

**wStr** GATGAAGCAC TTATTCCTCA CACTGCATTT ACTAC**G**TCA**G** TTGATGC**T**CG TTATGGTATT

wNo GATGAAGCAC TTATTCCTCA CACTGCATTT ACTAC**G**TCAA TCGATGC**T**CG TTATGGTATT

wBm GATGAAAAGC TTACTCCTCA TACTGCATT**C** ACTAC**A**TC**G**A TCGATGC**A**CG TTATGGTATT

* **** * ** *** *** *** *** * *** * *

301 360

wAna G**TTT**C**A**G**C**AG **AGGA**T**AGAAC** G**A**A**A**A**CT**A**TA** CAT**GCTGCTA** TCA**ACAA**GG**A** TG**G**T**ACTC**AG

wHa G**TTT**C**A**G**C**AG **AGGA**T**AGAAC** G**A**A**A**A**CT**A**TA** CAT**GCTGCTA** TCA**ACAA**GG**A** TG**G**T**ACTC**AG

wRi G**TTT**C**A**G**C**AG **AGGA**T**AGAAC** G**A**A**A**A**CT**A**TA** CAT**GCTGCTA** TCA**ACAA**GG**A** TG**G**T**ACTC**AG

wPip G**TTT**C**A**G**C**TG **AGGA**T**AGAAC** **AA**A**A**A**CT**A**TA** CAT**GTTGCTA** TT**GA**T**AAA**A**A** T**AGAACTC**AG

wAu **A**CAAC**A**GGTG TTTCTGCTCA TGATAGAACG CATACGATAC TTACTGC**C**AT TGATGA**A**AAG

wMel **A**CAAC**A**GGTG TTTCTGCTCA TGATAGAACG CATACGATAC TTACTGC**C**AT TGATGA**A**AAG

**wStr** **A**CAACGGGTG TTTCTGCTCA TGATAGAACG CATACGATAC TTAC**C**GCTAT TGATGA**A**AAA

wNo **A**CAACGGGTG TTTCTGCTCA TGATAGAACG CATACGATAC TTAC**C**GCTAT TGATGG**A**AAA

wBm GCAAC**T**GGTG TTTCTGCTCA TGATAGAACG CATACGATAC TTACTGCTAT TGA**C**GG**G**AAG

* * * * * *** * * *

361 420

wAna **GA**T**GAC**A**T**C**A** **TAAC**T**CC**A**GG** T**CA**TG**TTTT**T C**C**TGTA**A**TT**G** C**ACA**TA**A**AG**G** **TG**GGG**TG**GCA

wHa **GA**T**GAC**A**T**C**A** **TAAC**T**CC**A**GG** T**CA**TG**TTTT**T C**C**TGTA**A**TT**G** **CACA**TA**A**AG**G** **TG**GGG**TG**GCA

wRi **GA**T**GAC**A**T**C**A** **TAAC**T**CC**A**GG** T**CA**TG**TTTT**T C**C**TGTA**A**TT**G** C**ACA**TA**A**AG**G** **TG**GGG**TG**GCA

wPip **GA**T**GAC**A**TTA** **TAAC**T**CCCGG** T**CA**T**ATTTT**T C**C**TGT**GA**TT**G** C**ACA**T**GAG**G**G** **TG**GAG**T**AG**AG**

wAu AGTACTAA**G**G ACGATATTAT TACTCCAGGG CATGTTTTCC CTATTATTGC AAA**T**GAAGG**C**

wMel AGTACTAA**G**G ACGATATTAT TACTCCAGGG CATGTTTTCC CTATTATTGC AAA**T**GAAGG**C**

**wStr** AGTACTAA**G**G ATGATATTAT TACTCCTGGT CATGTTTTTC CTATTATTGC AAG**T**GA**G**GG**C**

wNo AGTACTAA**G**G ATGATATTAT TACCCCTGGT CATGTTTTTC CTATTATTGC AAG**T**GA**G**GG**C**

wBm AGTACT**C**A**A**G ATGATATTAT TACTCCTGGT CATGTTTTTC CTATTATTGC AAA**C**GA**T**GG**T**

* * * * * *** * * * * * *

421 480

wAna **CAACG**T**GCT**G **GT**C**A**TA**C**TG**A** **AGC**GA**G**TG**TT** G**A**AA**T**GG**C**G**A** A**GT**TAG**T**A**GG** GTT**T**G**A**T**CAT**

wHa **CAACG**T**GCT**G **GT**C**A**TA**C**TG**A** **AGC**GA**G**TG**TT** G**A**AA**T**GG**C**G**A** A**GT**TAG**T**A**GG** GTT**T**G**A**T**CAT**

wRi **CAACG**T**GCT**G **GT**C**A**TA**C**TG**A** **AGC**GA**G**TG**TT** G**A**AA**T**GG**C**G**A** A**GT**TAG**T**A**GG** GTT**T**G**A**T**CAT**

wPip **CAACG**T**GCT**G **GT**C**A**TA**C**TG**A** **AGCA**A**G**TG**TT** G**AG**A**TA**G**C**T**A** A**GT**TAG**TGGG** ATG**T**A**A**T**CAT**

wAu GGGGTTTTAG CACG**C**AATGG TCA**C**ACTGAA GCAAGTGTTG AAATAGCAAA GTT**G**GTTGG**T**

wMel GGGGTTTTAG CACG**C**AATGG TCA**C**ACTGAA GCAAGTGTTG AAATAGCAAA GTT**G**GTTGG**T**

**wStr** GGGGTTTTAG CACG**C**AACGG TCATACTGA**G** GCAAGTGTTG A**G**ATAGCAAA ATT**G**GTTGGA

wNo GGGGTTTTAG CACGTAACGG TCATACTGAA GCAAGTGTTG A**G**ATAGCAAA ATT**G**GTTGGA

wBm GGGGTTTTAG CACGTAATGG TCATACTGA**G** GCAAGTGTTG AAATAGCAAA GTT**A**ATTGG**C**

* * * * * * ** * * * * **** ** *

481 540

wAna T**C**A------- --GC**A**GT**TAT T**TGTGAA--- TTAGTGAATG ATGATGGCTC TATGATGCGC

wHa T**C**A------- --GC**A**GT**TAT** **T**TGTGAA--- TTAGTGAATG ATGATGGCTC TATGATGCGC

wRi T**C**A------- --GC**A**GT**TAT** **T**TGTGAA--- TTAGTGAATG ATGATGGCTC TATGATGCGC

wPip T**C**T------- --GC**A**GT**TAT** **T**TGTGAA--- GT**G**AATGATG ATGGCTCTAT GATGCGCTT**A**

wAu CTTAATCATG CAGCTGTAGG GTGTGAATTA GT**G**AATGATG ATGGCTCTAT GATGCGCTT**A**

wMel CTTAATCATG CAGCTGTAGG GTGTGAATTA GT**G**AATGATG ATTGCTCTAT GATGCGCTT**A**

**wStr** T**C**TAATCATG CGGCTGTAGG GTGTGAG**C**TA GT**G**AATGATG ATGG**A**TCTAT GATGCGCTT**A**

wNo T**C**TA**G**TCATG CGGCTGTAGG GTGTGAATTA GT**G**AATGATG ATGGCTCTAT GATGCGCTT**A**

wBm TATAATCATG CAGCTGTAGG GTGTGAATTA GTAAATGATG A**C**GGTTCTAT GATGCGCTT**G**

* ***** * ** ** ****** ** * *** * * ***

541 600

wAna TTACCTCAGT TACTTAAATT TGCTGAACAA CATAAAATTA AGTTAACTAC CAT**C**GAC---

wHa TTACCTCAGT TACTTAAATT TGCTGAACAA CATAAAATTA AGTTAACTAC CAT**C**GAC---

wRi TTACCTCAGT TACTTAAATT TGCTGAACAA CATAAAATTA AGTTAACTAC CAT**C**GAC---

wPip **CCCCAGTT**G**C** T**TGAATTTGC** TG**AAC**AACAT **A**ATA**TT**A**AGT** **TAACT**AC**C**A**T** T**GA**T**A**A**A**---

wAu TTA**C**CTCAGT T**G**CTTAAATT TGCTGAACAA CATAAAATTA AGTTAACTAC CAT**C**GACAAA

wMel T**C**T**ATGAT**G**C** **GCT**T**ACCTCA** **GTTGCTTA**AA **TT**T**GCTGA**A**C** AA**CAT**A**AA**A**T** TA**AGTTA**---

**wStr** **CCCCAGTT**G**C** T**TGAATTTGC** TG**AAC**AACAT **A**ATA**TT**A**AGT** **TAACT**AC**C**A**T** C**GACA**A**A**---

wNo **CCCCAGTT**G**C** T**TGAATTTGC** TG**AAC**AACA**C** **A**A**A**A**TT**A**AGT** **TAACT**AC**C**A**T** C**GA**T**A**A**A**---

wBm **CC**T**GAATT**G**C** T**TAAATTTGC** TG**AAC**AACAT **A**ATA**TT**A**AGT** **TAACT**AC**C**A**T** C**GA**T**A**A**A**---

* * *

601

wAna CTTATCAGTT ACGTTAAAAA TTTAAACTAG

wHa CTTATCAGTT ACGTTAAAAA TTTAAACTAG

wRi CTTATCAGTT ACGTTAAAAA TTTAAACTAG

wPip CTTATCAGTT ACGTTCAAAA TTTAAACTAG

wAu CTTATCAGTT ACGTTAAAAA ATTAAACTAG

wMel CTTATCAGTT ACGTTAAAAA ATTAAACTAG

**wStr ---------- ---------- ----------**

wNo CTTATCAGTT ACGTTCAAAA TTTAAACTAG

wBm CTTAT**AC**G**C**T AC**A**TTAAAAA A**C**TAAGCTAG

***** * * ** ** **** ********

**Figure S3.** Nucleotide sequence alignment of *rib*B homologs from ^B^*w*Str and WOL-A, B- and D-strains at left in red, blue and black font, respectively. Asterisks below the alignment indicate universally conserved nucleotides. Unique nucleotides are in green font. Nucleotides conserved in ^B^*w*Str and a majority of B-strains are in dark blue bold font, while those in dark red bold font are conserved with a majority of A-strains. Nucleotides conserved in two to four strains are in light blue, orange or orange bold font. See Tables 2 and S2 for host associations and Genbank Accessions.
